# Awareness and Learning in Participatory Noise Sensing

**DOI:** 10.1371/journal.pone.0081638

**Published:** 2013-12-11

**Authors:** Martin Becker, Saverio Caminiti, Donato Fiorella, Louise Francis, Pietro Gravino, Mordechai (Muki) Haklay, Andreas Hotho, Vittorio Loreto, Juergen Mueller, Ferdinando Ricchiuti, Vito D. P. Servedio, Alina Sîrbu, Francesca Tria

**Affiliations:** 1 Complex Networks and Systems Lagrange Laboratory, Institute for Scientific Interchange Foundation, Turin, Italy; 2 Extreme Citizen Science Research Group, Department of Civil, Environmental and Geomatic Engineering, University College London, London, United Kingdom; 3 Department for Artificial Intelligence and Applied Computer Science, University of Würzburg, Würzburg, Germany; 4 Physics Department, Sapienza University, Rome, Italy; 5 CSP Innovazione nelle ICT, Turin, Italy; 6 Department of Electrical Engineering/Computer Science, University of Kassel, Kassel, Germany; 7 L3S Research Center, Leibniz Universität, Hannover, Germany; US Naval Reseach Laboratory, United States of America

## Abstract

The development of ICT infrastructures has facilitated the emergence of new paradigms for looking at society and the environment over the last few years. Participatory environmental sensing, i.e. directly involving citizens in environmental monitoring, is one example, which is hoped to encourage learning and enhance awareness of environmental issues. In this paper, an analysis of the behaviour of individuals involved in noise sensing is presented. Citizens have been involved in noise measuring activities through the WideNoise smartphone application. This application has been designed to record both objective (noise samples) and subjective (opinions, feelings) data. The application has been open to be used freely by anyone and has been widely employed worldwide. In addition, several test cases have been organised in European countries. Based on the information submitted by users, an analysis of emerging awareness and learning is performed. The data show that changes in the way the environment is perceived after repeated usage of the application do appear. Specifically, users learn how to recognise different noise levels they are exposed to. Additionally, the subjective data collected indicate an increased user involvement in time and a categorisation effect between pleasant and less pleasant environments.

## Introduction

Public participation in environmental decision making was pushed to the fore as a result of the Rio Declaration on Environment and Development [1]. However, the provision and production of environmental information, particularly on issues such as noise pollution and air quality, rely heavily on a ‘top-down’ approach in which public authorities collect the data and release it to the public. There is still room to develop better mechanisms that support citizens to not only consume but to generate their own environmental information. If successful, such processes could lead to an increased awareness and learning about current environmental issues. Furthermore, this may serve to encourage more citizens to participate in environmental decision making, and ultimately stimulate them to take steps to improve their own environment based on new observation techniques.

Noise pollution is a problem in cities across the world and is one that is likely to affect an increasing number of people with the majority of the global population now living in urban areas [2]. In Europe, this has been recognised and abatement measures have been introduced in many countries. However, noise pollution, in particular, is an environmental problem that relies heavily on ‘top down’ approaches, both in terms of communicating the issue, through instruments such as strategic noise maps, but also in the methods used to gather data. For example, strategic noise mapping became a requirement of all Member States under the EU's European Noise Directive (ENDS). The maps are used to estimate population exposure to noise in certain areas, to communicate to the public and as a basis for action plans [3].

Exposure to noise is not merely a case of annoyance. Researchers have provided a growing body of evidence that suggests that long-term exposure to noise constitutes a health risk hazard and can modify social behaviour, cause annoyance [4], increase the risk of cardiovascular diseases [5] and adversely affect levels of attentiveness and the ability to read in children [6]. The World Health Organisation (WHO) estimated that at least one million healthy life years are lost every year from traffic-related noise in the western part of Europe [7].

New participatory sensing applications that exploit information and communication technologies (ICT) are providing novel approaches to environmental monitoring. Simultaneously, they present an opportunity to widen citizen engagement and participation in local, regional and global environmental issues. This has been enabled, in part, by the relative affordability and growth in the number of smartphones in use, now estimated to have breached the 1 billion mark [8]. Miniaturisation of embedded sensors in these devices, such as a microphone, camera, accelerometer, and GPS receiver, combined with the increasing computation power, network connectivity and data plans has resulted in an increasing number of smartphone Apps (short for applications) designed for a range of participatory sensing opportunities.

Participatory sensing, also referred to as urban sensing, involves enabling individuals, groups and communities to gather, document, view, share, and in some cases analyse local observations and data about their surrounding environment. Not all participatory sensing relies on mobile technologies. For example, [9] comment on the use of low cost noise monitors in a citizen science project in which two communities collected noise data: one in relation to noise nuisance being generated by a local scrap yard and the other, in an objection to an airport expansion plan. However, the use of smartphones as sensory devices, either passively or actively, increases the ability to scale such activities. Cuff et al. [10] highlight a range of applications in which citizens can be engaged in mobile sensing, predicting a growth in the field and in the numbers of ways in which it will be applied.

The power of the ‘crowd’ has been recognised as an effective way of generating observations, which might otherwise be difficult to obtain, due to spatial and temporal limitations. This is particularly relevant in fields where traditional sensing relies either on a distributed network of expensive stationary monitoring devices across a target area of interest, or where sensors require physical placement for a specific deployment, or in cases where numerical simulations are needed. Cost and data coverage are key factors. The spatial distribution of static monitoring devices and the associated costs of hiring trained specialists to take measurements and process data reduce the amount of real-world measurements that can be taken.

Noise provides a good example for this problem, and is one of the environmental domains in which computer simulation models are used to predict noise exposure from various sources such as different modes of transportation in a given area. Measurements are often used to validate the results. However, observations and data provided through participatory sensing activities could provide added value to existing data sources.

Applications such as *NoiseWatch* (EEA) in which the phone's microphone is used to determine the level of sounds it detects, which is then displayed in decibels (dB), have begun to emerge [11]. An earlier example is *NoiseTube*, which adopts a passive approach to noise monitoring. The App constantly collects environmental sound, in second-long samples, provided that the application is left running [12]. An additional feature allows users to annotate and tag measurements. The *Noise Nuisance* App, created by a group of environmental health professionals, provides a country specific design that is tailored to tackle noise nuisance in the UK. It provides users with relevant email addresses of every local authority noise investigation team which is programmed into the App, information on statutory noise, and alternative action measures that can be adopted. Users can record audio samples, make an entry into a diary each time the noise affects them and report these to their environmental health department or housing association officer [13].

All these applications are providing the tools to record the noise, with some attempting to overcome the technical limitations of smartphone and produce more accurate measurements. Other than a preliminary analysis of tagging patterns in the NoiseTube community [14], the authors are not aware of any studies that have provided empirical data that explores user behaviour/opinion patterns over time. More specifically, that points to the change of user opinions/awareness of perceived noise levels that may occur with an increase in use of such applications.

Here, we present results from participatory sensing using the *WideNoise* application (recently renamed as *WideNoisePlus*). This is a smartphone application developed within the EveryAware project [15], which was designed not merely as a measurement tool for its users, but also as a means to monitor opinions on the environment and noise, in a way as transparent to the user as possible. Hence the application has several features that allow for subjective/personal data to be acquired. Using these data, an analysis of user behaviour/opinions that may emerge after usage of WideNoise will be performed. Changes in behaviour are indeed visible after a user performs several measurements, which is a strong indication of increased awareness and learning.

## Methods

The work presented here is part of the European project Every Aware, contract number IST-265432. The European Commission finances only those projects that comply to its ethics and privacy regulations. Citing from the regulations of the Seventh Framework Programme, Decision No 1982/2006/EC[16], Article 6: “All the research activities carried out under the Seventh Framework Programme shall be carried out in compliance with fundamental ethical principles.” At the same time, the official rules for participation[17], Article 15, mention: “A proposal […] which contravenes fundamental ethical principles […] shall not be selected. Such a proposal may be excluded from the evaluation and selection procedures at any time.”

Hence, acceptance and funding of this work by the European Commission implies approval of the ethics statement made in the proposal. This is why no further formal ethics approval was required for this research to be performed. This includes participants from outside the European Union (since the project clearly stated that a publicly available mobile App will be developed).

All participants to our study had to install the mobile application in order to perform measurements. Upon installation, all users were required to accept the Terms and conditions[18] of the app, which represents the user's consent to use the measurements made. Unacceptance rendered the installation process impossible. The full terms and conditions clearly state that the data will be used for research purposes only and no personal information will be made public or used for other purposes.

### WideNoise platform - Noise sensing

WideNoise is a mobile application for recording, monitoring and analysing noise pollution. The application is intended to run on mobile devices and more specifically on Android[19] and iOS[20] platforms. It was originally developed by WideTag[21] and then was enhanced by the EveryAware team, who has improved and expanded the data recorded from the mobile device by adding new features. The mobile application sends anonymous data to an application server capable, through RESTful web services, of collecting the acquired data and showing the corresponding information on a map (details in Supplementary Material S1). Both sensor data and subjective perceptions are required to create a full sound report, so that the application consists of two main parts: the noise sampling component and the perception tagging.

**Figure 1:**
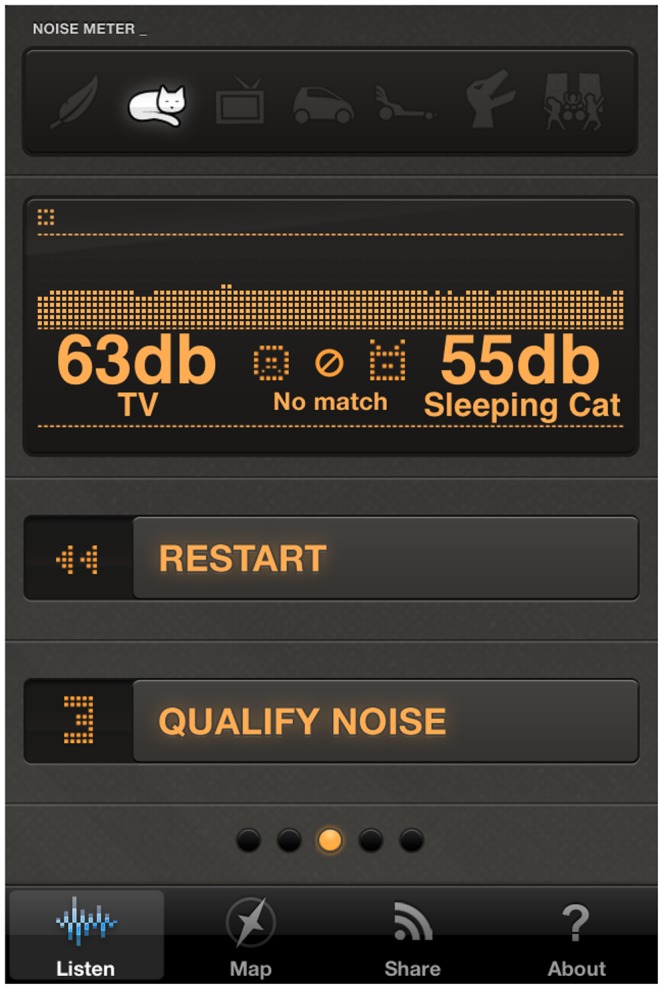
Noise sample screen for WideNoise Plus mobile application.

The noise measurement part gives users the possibility to take a noise sample through the smartphone microphone. When the recording starts, the user is asked to guess the noise level through a slide bar where a decibel scale is mapped. The user has also the possibility of extending the default sampling time. In this way, while the user gets more time to make the guess, the app will perform a longer measurement. After the recording phase, the noise level expressed in decibels (dB), is shown and compared to the level estimated by the user. The sound level is associated with an iconographic representation that allows the user to better understand the decibel value, by using seven intuitive noise sources: falling feather, sleeping cat, tv show, car engine, dragster, t-rex and rock concert (Figure 1).

**Figure 2:**
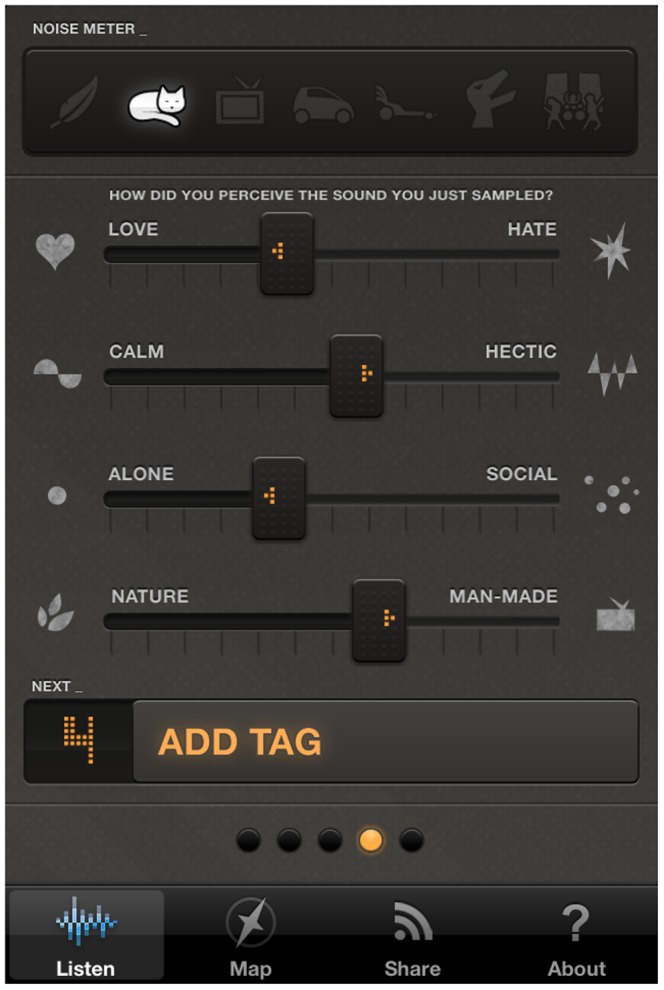
Perception screen for WideNoise Plus mobile application.

**Figure 3:**
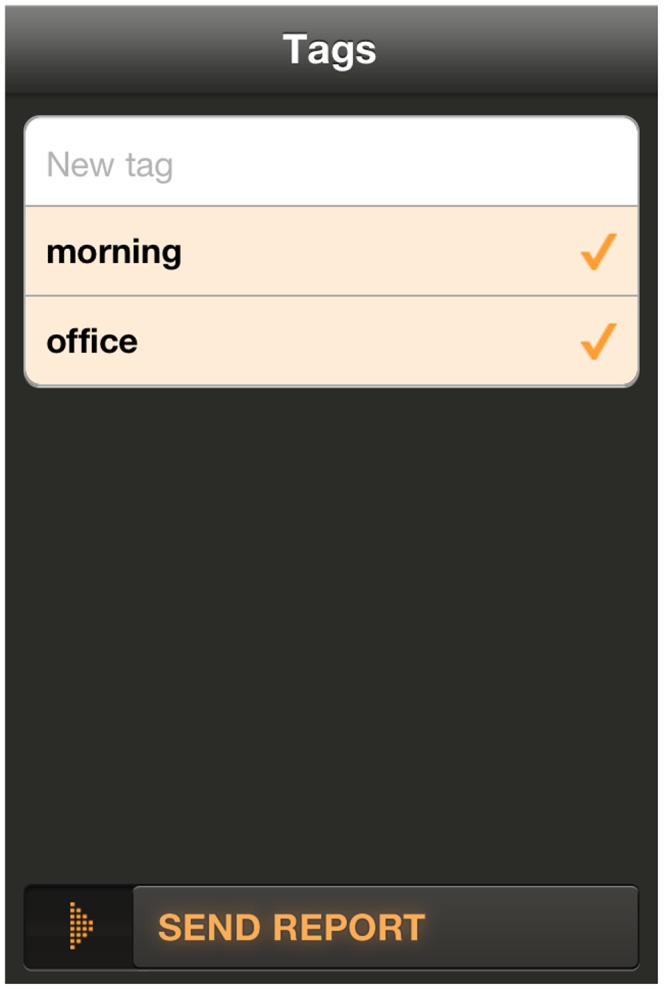
Tag screen for WideNoise Plus mobile application.

**Figure 4:**
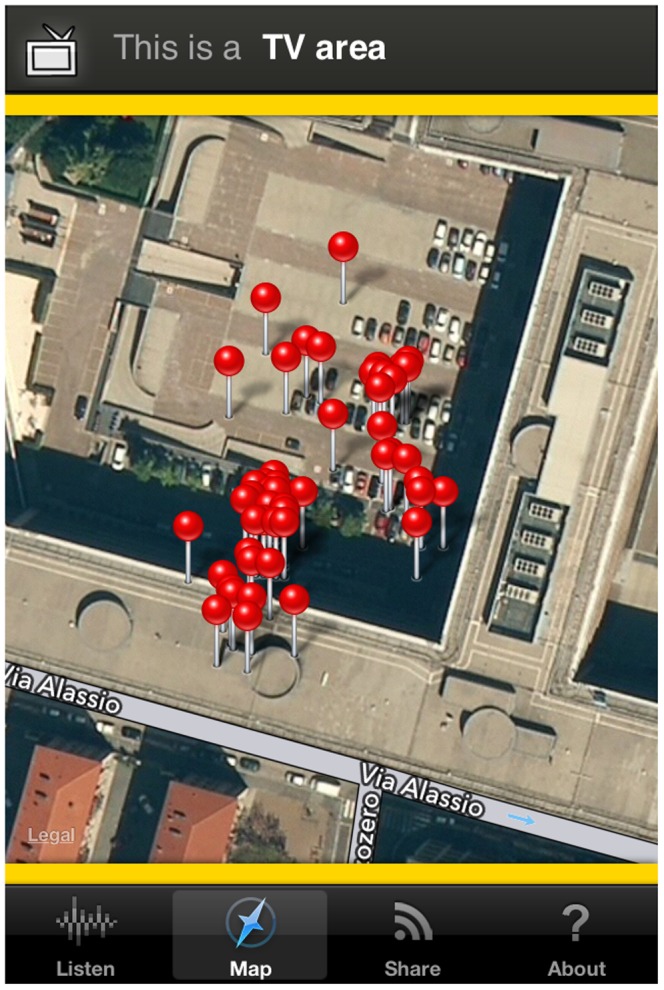
Map screen for WideNoise Plus mobile application.

After the noise recording/guessing stage, the users are asked to express their own feelings about their current environment. They can provide a score by moving four different sliders associated to as many feelings and categories: love/hate, calm/hectic, alone/social, nature/man-made (Figure 2). The users can also associate free text tags to the noise. Once the tags are applied, all the information collected by the application is sent to the web application server as soon as a working data connection is available (Figure 3). WideNoise allows users to view a community map displaying the average noise level at nearby locations, by relying on the statistical elaboration provided by the server (Figure 4). As an integration with social networks, users can also share their own recordings via Twitter and Facebook.

### Participation

An obvious key integral component to any participatory sensing activity is the participants (and users). The EveryAware project has stated a clear goal to enable public participation in sensing activities and as such, face-to-face and virtual communication was adopted to recruit participants for the initial test cases. Three public test cases were initiated covering the period from February, 2012 to May 28th 2013. The first occurred during the Citizen CyberScience conference in London, which ran over a period of three days. Around 170 delegates were encouraged to download the WideNoise App and to take measurements in the conference facilities and in the surrounding area. The second test case focused on engaging communities surrounding London Heathrow Airport and was kicked-off by a launch event in the Isleworth community on the 19th of June 2012. In 2013, the test case was extended to the community of Windsor, with WideNoise adopted by the local authorities and a training session organised by our team on the 23rd of April. The third test case involved a one-day event held at a bookshop in Rome (9th June, 2012). Visitors to the bookshop were encouraged to capture noise measurements from the surrounding streets and these were visualised in real time on a large screen inside the store. Additionally, an internal test case was also organised in Antwerp, Belgium, on the 10th of July 2012, where members of the EveryAware team performed measurements in the city centre area. Also, a workshop with architecture students was held in Birmingham on the 5th of October 2012.

For the Citizen CyberScience conference an email was sent to all the delegates prior to the start of the conference. Email reminders were also sent every morning over the course of the three days. In addition, a short presentation was given by one of the EveryAware team members on the first day inviting people to participate. Custom business cards with links to the WideNoise application were handed out to all the delegates and project team members were on-hand to answer questions.

In order to recruit communities surrounding London Heathrow we adopted a ‘hands on’ approach, as well as a ‘virtual’ approach. Virtual recruitment included banner ads on hyper-local websites in addition to posting on Twitter and Facebook. Articles were published in several local papers and the project received coverage on BBC London news and local radio. Hand-distributed flyers were circulated and posters were placed in shop windows. The mailing list of an anti-airport expansion campaign organisation was used to inform people about the project. An official launch was held in a local community centre that was attended by local residents, a local councillor and members of the campaign group.

The third test case in Rome was promoted via Twitter, Facebook and other online web sources, as well as having a dedicated website[22]. It was also advertised in print media throughout the locality in the days before the event in order to attract participants from the neighbourhood. Several posters and flyers were placed in areas commonly frequented by local residents.

### Awareness indicators

Although in itself a tool for measuring noise, the WideNoise application contains several features that allow the users to share their opinions on the environment. This makes it an interesting tool for studying changes in behaviour/perception and learning, very important aspects when dealing with environmental issues. Awareness and learning indicators have been derived from these data, and used to build the discussion in the Results Section.

One of these features, as already anticipated, is that the users can try to guess the level of noise around them, while the sampling procedure itself is still running. This appears as a game to the player and makes the application more interesting, generating at the same time additional data. In this paper, we are interested in how accurate the user estimations are, and more importantly, in how the accuracy does change in time, after several measurements are performed. An increased accuracy in time would indicate that users are learning from the application, becoming in a sense human sensors.

A different feature that allows for subjective data to be acquired is the possibility to add perception ratings (Love-Hate, Calm-Hectic, Alone-Social, Nature-Man made) on a scale from 0 to 1. These indicate how users perceive the environment, having also available the information about noise levels just measured. It is interesting to see how this perception changes after repeated use of the application, and whether signs of increased awareness are observed.

Furthermore, users can share other custom information as well, in the form of tags. While studying the texts of the tags can give semantic content and in particular useful insight into the locations that users are interested to measure, here we will again focus on analysing awareness. The tagging procedure is more time costly compared to the other two features mentioned above. Hence tagging is a strong indication of a user being committed to the task, being aware of the problem around and trying to document it in some way. Our study will investigate how the tagging behaviour changes after multiple measurements are performed. We are interested in the fraction of measurements that are tagged for each user. If this grows in time, then the user most likely has become more dedicated to the task, which is a sign of increased awareness.

## Results and Discussion

### Participation and coverage

Although the aim of this paper is the study of learning and awareness in participatory sensing, it is important to define the structure of the data collected, to support the results obtained. In this section we will give a general description of the amount of data, participation patterns and coverage, with further details on more specific patterns and individual test cases included in Supplementary Material S1.

**Figure 5:**
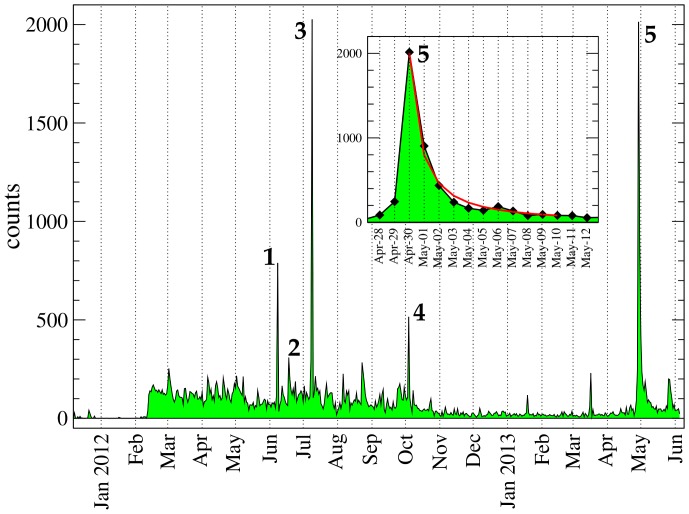
Measurements per day. Number of measurements collected each day from Dec. 8th 2011 till Jun. 6th 2013. The labels correspond to: (1) case study in Rome (9th June 2012); (2) launch of the Heathrow activities (19th June 2012); (3) Antwerp test case (10th July 2012); (4) Birmingham workshop (5th October 2012); (5) article in German regional newspaper (published 29th April 2013, activity peak on the 30th of April 2013). In the inset an enlarged view of event 5 is showed. The decay of user participation is consistent with a power-law of exponent 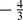 (red curve).

The data considered for analysis have been collected up to June 7th 2013 at 3AM. These consist of 41478 sound level measurements made by 13962 unique devices. Figure 5 shows the number of measurements collected each day since December 2011. The higher spikes correspond to case studies or public advertising of our application. For instance, the first two spikes marked **1** and **2** correspond to the test case in Rome (9th June 2012) and to the launch of the Heathrow activities (19th June 2012) respectively. Another activity peak can be observed on the 10th of July, marked as **3**, corresponding to the Antwerp test case, while the peak number **4** on the 5th of October 2012 corresponds to the workshop organised in Birmingham. The activity in 2013 is boosted by the publication of an article in a German regional newspaper on the 29th of April that triggered a substantial measuring activity all over Germany, generating also a large peak on the 30th of April, labelled with number **5**. Given that this last activity spike was not produced by an organised test case, it is interesting to study the trend around it in detail. The inset in Figure 5 shows a possible power-law decay with exponent around −4/3 of the number of measurements in time. This means that the general interest is dropping fast, however several users maintain their activity for a longer time. Such power-law decay of human activities has been already reported for the occurrences of words related to special events in Japanese blogs [23]. In their case the power-law decay of extreme events broadcast by media (eg. *Tsunami*) was estimated with an exponent of 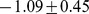. Moreover, the power-law behaviour we find, seems to be well described by the exogenous subcritical process described in Ref. [24].

**Figure 6:**
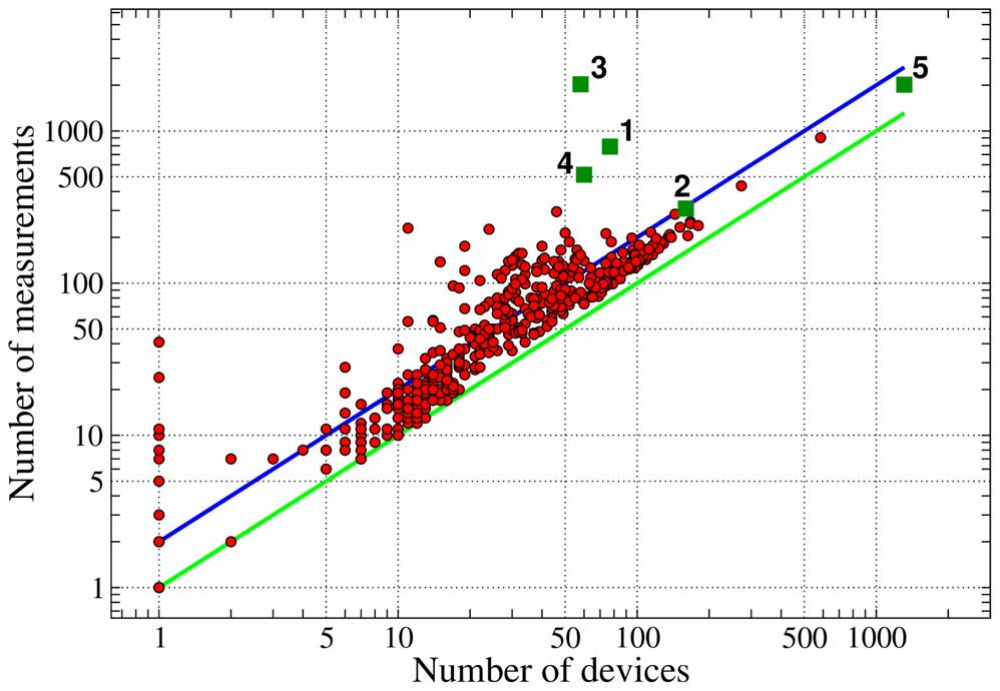
Measurements versus devices. Scatter plot of the number of measurements collected each day compared to the number of active devices at that day. The dark green symbols correspond to the most important spikes shown also in Figure 5. The green and blue lines are guides for the eye and correspond to the case of one measure per device and two measures per device respectively.

For more details on how these activities were generated, Figure 6 shows the number of contributing devices for each day, compared to the number of measurements. The points corresponding to the peaks observed in Figure 5 are clearly marked. In general, there appears to be a linear dependence between the number of devices per day and their corresponding measurements. The peaks corresponding to the Heathrow start of activity (marked 2) and the newspaper article (marked 5) fit within the linear dependence, due to the involvement of a larger amount of citizens in the measurements. Each device performs less than 2 measurements per day on average. Other test cases, such as the internal one in Antwerp, the one in Rome or the workshop in Birmingham (1, 3 and 4), did not attract as many users. However a large amount of measurements have still been obtained, shown by the deviation of the corresponding points from the larger mass of measurements. Here, each device performs much more measurements than in the previous case.

Thus, when analysing the days with higher number of measurements, we can distinguish between two types of activity trends: (i) large amounts of data generated by a small set of motivated users (peaks 1, 3 and 4) and (ii) large amounts of data generated by a correspondingly larger user base (peaks 2 and 5). The difference between the two types of behaviour can depend on several factors. For (i) the activities were goal-oriented and lasted for only one day. Users were asked to make an effort on the day with no incentives, except for the case study in Rome where the first three users with most measures were rewarded with a low value book gift voucher. This explains the large one-day activity per person. However, for the Heathrow case study as well as for the newspaper article, activities were not limited to a single day. Users did not have to concentrate all their measurements in a few hours, but were free to take samples over a longer period. It appears from the data that the natural average density for a single user is of two measurements per day. Additionally, the two types of activity trends were caused by different incentives. The activities in the first category were dedicated to covering as much area as possible and obtain a map of the daily noise. In the newspaper case, on the other hand, no incentive was given. Users were just measuring interesting noise levels. This is an event-based scenario that seems to favour a dilution of the measurements over multiple days.

**Figure 7:**
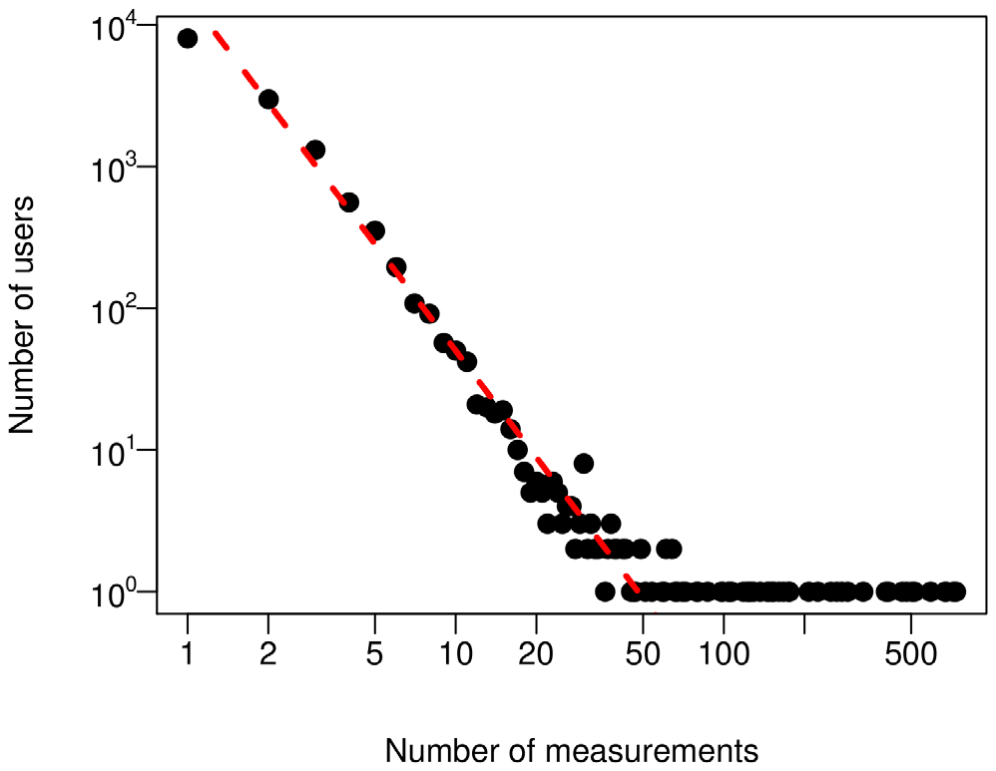
User activity distribution. Power-law compatible distribution of the number of measurements performed by each user. The red dashed line corresponds to a powerlaw of exponent −2.5.

General user activity patterns have been also studied. Figure 7 displays the distribution of the number of measurements submitted by individual users. This appears to be consistent with a power-law, with a large number of users submitting a small amount of measurements and, conversely, a small number of users performing a very large amount of measurements. This behaviour is the footprint of social activity, with the power-law distribution appearing in many other settings, for instance social network activity measures [25]. Hence the WideNoise user activity fits very well in the general known patterns of human behaviour.

**Figure 8:**
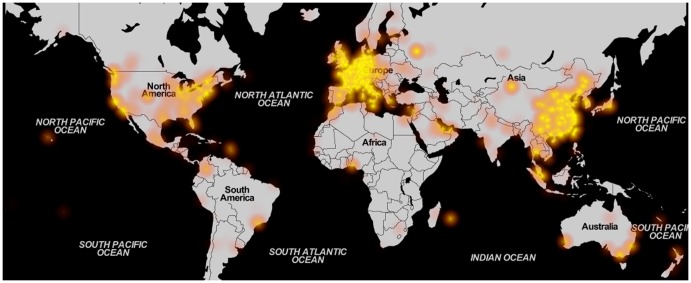
Overall heatmap. Worldwide sample density, including all measurements, illustrated as a heatmap (© *OpenStreetMap contributors* for map data, used and redistributed under the CC-BY-SA licence[26]).

While noise measurements can be performed with static monitors or by official agencies, distributed mapping of noise has the advantage of yielding a wider coverage in time and/or space. The data collected by WideNoise users come from across the world, with some areas better represented and covered than others. The general pattern is visualised in Figure 8, while Table 1 shows coverage indicators for the data acquired, both worldwide and at continent level. Details for smaller areas of interest, corresponding to the different test cases organised, can be found in Supplementary Material S1. The indicators shown in the table are the number of measurements, the average noise level, space and time coverage and the number of devices. Space coverage is computed by dividing the surface of interest into square tiles with the side of 15 meters. Any tile is considered covered if there is at least one measurement inside the corresponding square. Time coverage considers 30 seconds before and after the time of each measurement (i.e. one minute per data point), with overlapping regions removed. The table indicates Europe and Asia as the most active areas, with a few square kilometres and hundreds of hours of measurement covered. While the large amount of data for Europe is understandable, since it is the area where all the test cases have been organised, the Asian activity stands out as emerging without any intervention from the researchers' side. Regarding the average noise levels recorded, Europe, South America and Africa display higher noise values compared to the worldwide average (however the amount of points acquired in South America and Africa is very low). Asian and Australian users, on the other hand, report lower noise compared to the average. The difference between Asia and Europe (the two most active continents) could be again due to the fact that most test cases, organised in Europe, have concentrated on documenting bothersome high levels of noise in residential areas, leading to higher averages overall. This indicates a difference in the distribution of noise levels for measurements obtained in an organised versus an ad-hoc setting.

**Table 1:** General space and time coverage.

Location	Number of measurements	Area covered (km^2^)	Total Time (hours)	Average noise level (dB)	Devices
World	40661 (817 without location)	4.80982	541.93	64.16	13962
Europe	27771	3.36757	354.8	65.98	7395
Asia	11033	1.1358	164.49	59.59	5392
North America	1373	0.232655	21.59	64.39	588
South America	93	0.015525	1.51	66.25	56
Africa	107	0.01597	1.70	67.42	47
Australia	193	0.02610	3	58.44	96

**Figure 9:**
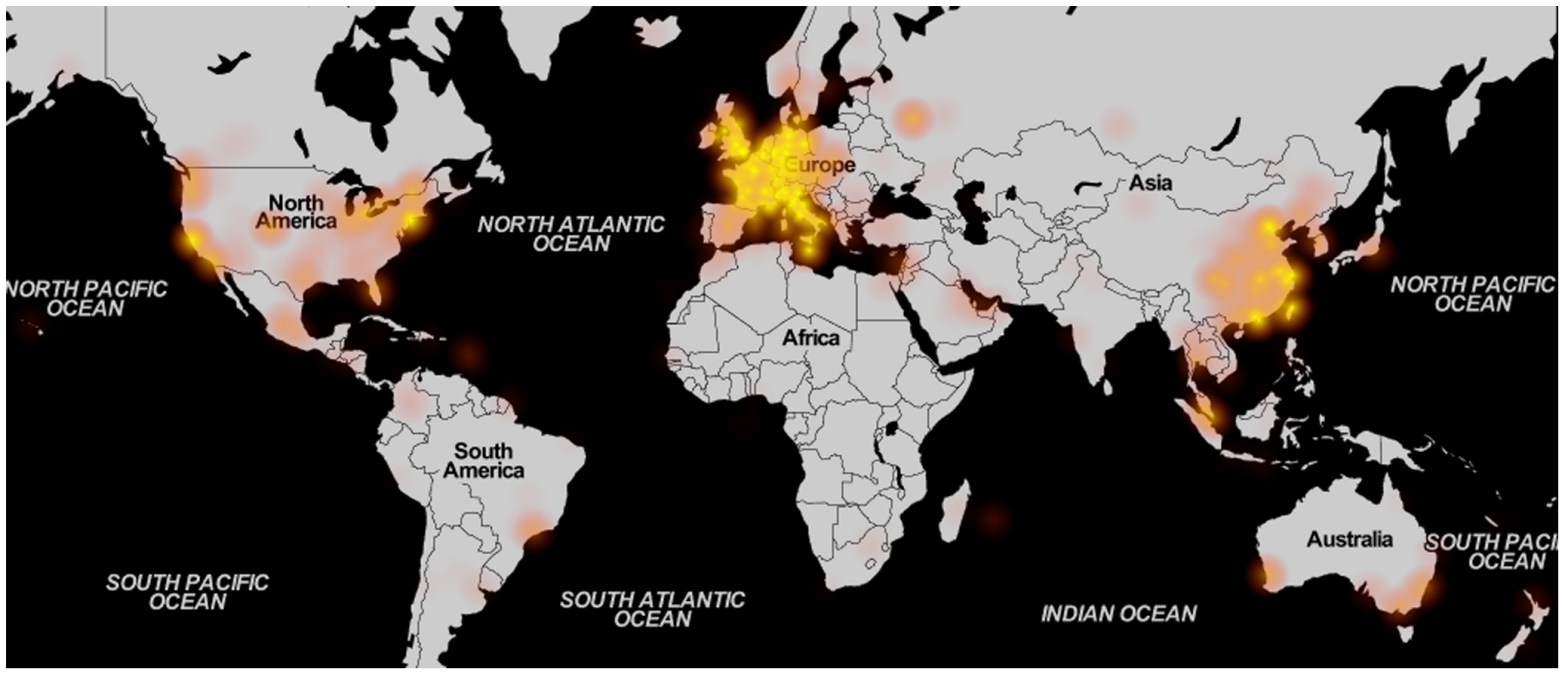
Perception heatmap. Worldwide sample density, including only measurements with attached perceptions, illustrated as a heatmap (© *OpenStreetMap contributors* for map data, used and redistributed under the CC-BY-SA licence[26]).

**Figure 10:**
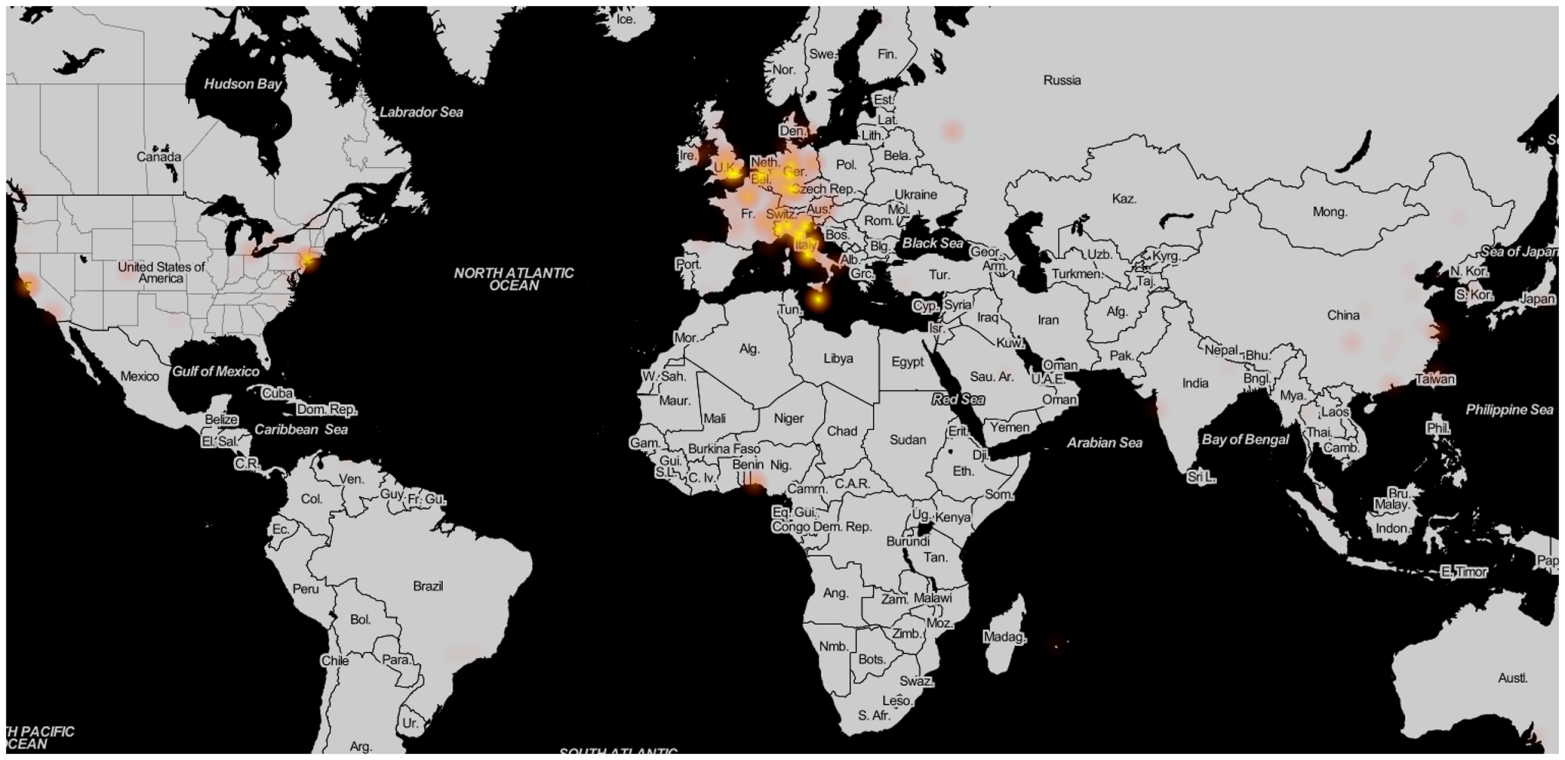
Tag heatmap. Worldwide sample density, including only measurements with attached tags, illustrated as a heatmap (© *OpenStreetMap contributors* for map data, used and redistributed under the CC-BY-SA licence[26]).

In order to assess awareness and learning, the subjective data submitted by users are very important. However, not all measurements contain the additional data (tags, perception annotation). Figures 9 and 10 show the distribution across the world of perception and tag data (details in Supplementary Material S1). These show that, compared to the overall density, the subjective data is reduced, especially concerning tag usage. For instance, while a very large number of measurements come from Asia, the majority have no tags attached (probably because in the App the western keyboard is displayed). Perception rating, on the other side, seems to be more uniformly distributed among the overall samples.

### Awareness indicators

By means of the subjective data collected during measurements, an analysis of user awareness will be presented in the following. The interest is in assessing whether usage of the application leads to any change in behaviour, and whether this change indicates an increase in awareness of environmental noise and its effects. For this study, only data collected by users not belonging to the EveryAware consortium is considered (38267 measurements).

**Figure 11:**
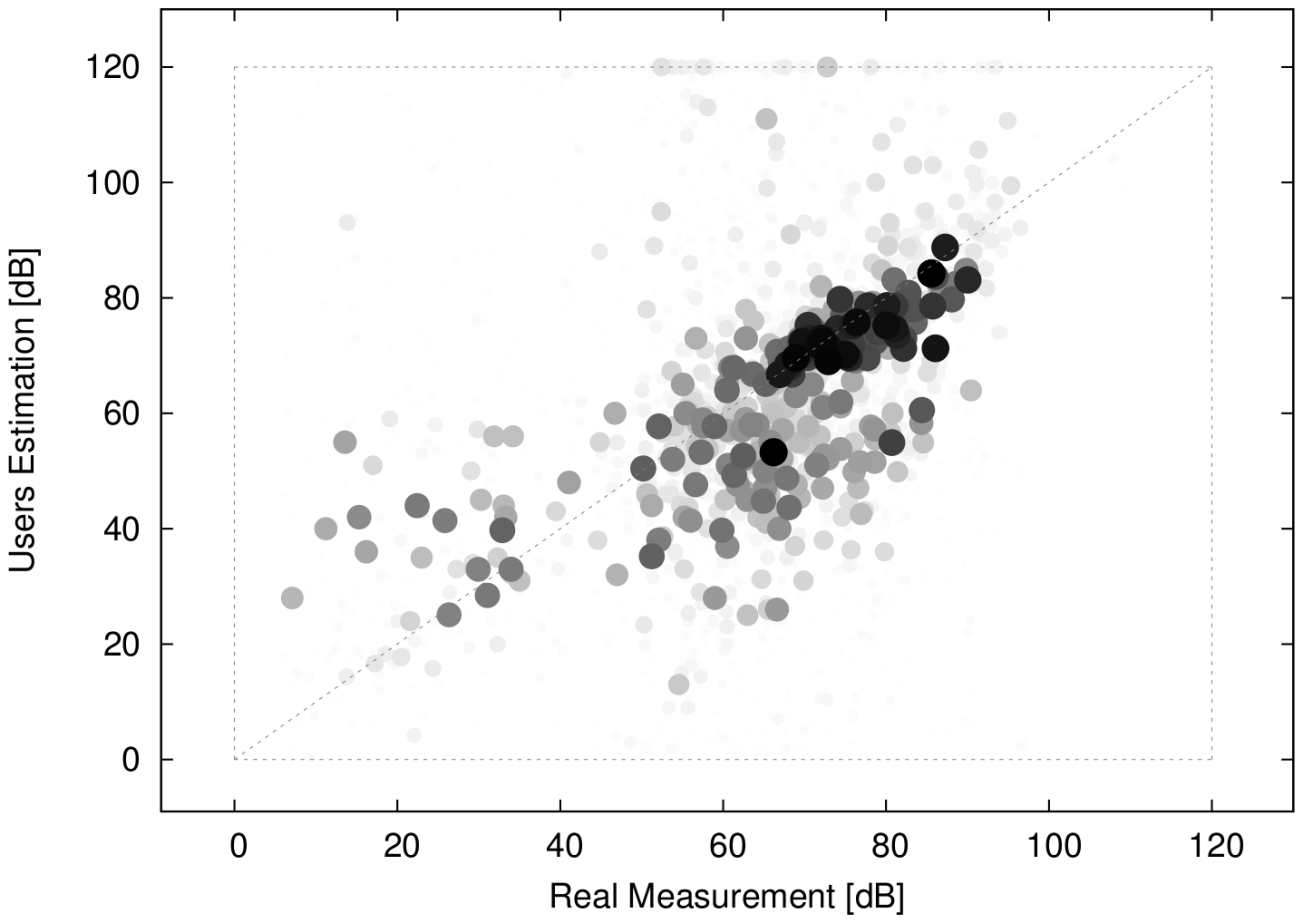
Estimated versus measured noise. Each point corresponds to one measurement, while both the colour scale light to dark grey and the point size represent the user expertise (small to large amount of previous measurements).

A first analysis of awareness/learning involves studying the decibel values estimated by users, in comparison with the measured values. Figure 11 displays the estimated vs real noise level, with light-coloured small points corresponding to early measurements by a single user, while dark large points corresponding to later measurements. Hence, the size and darkness of points displays user expertise. The figure shows larger darker points closer to the diagonal compared to lighter ones, which means that the estimation is closer to the measured value for later measurements. This indicates that during repeated usage of the application the ability of users to guess the noise level around them increases, hence the user learns in time.

**Figure 12:**
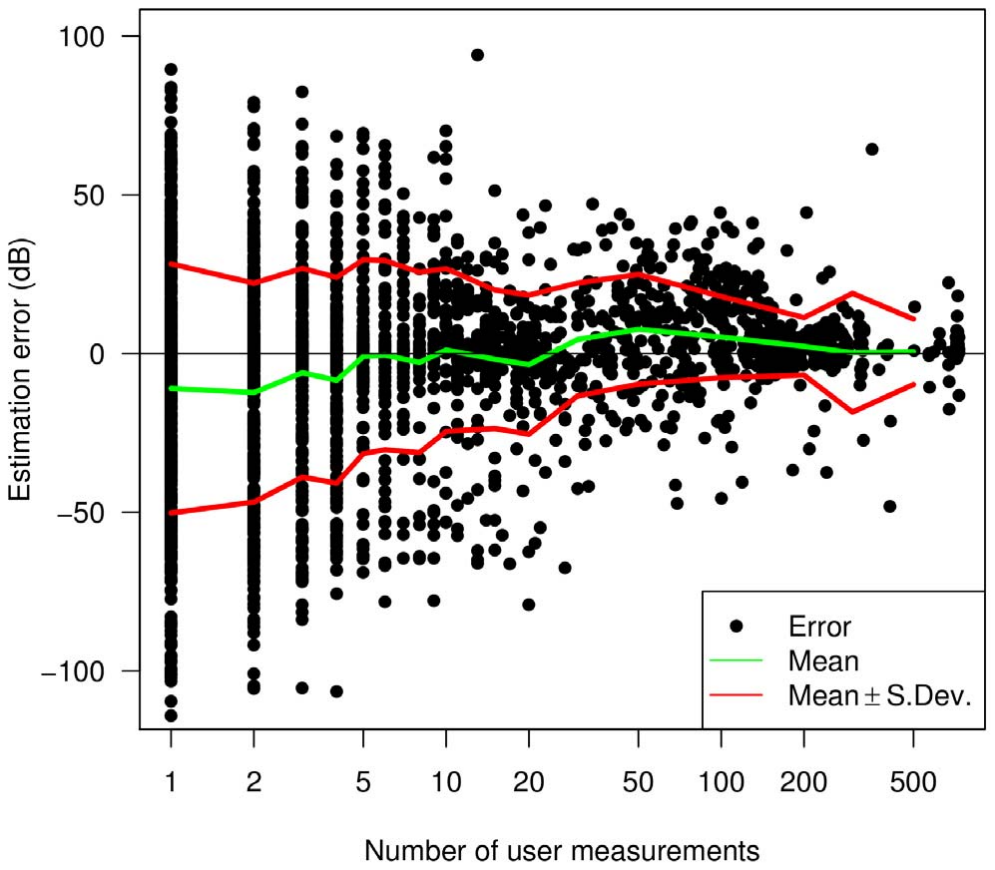
Estimation error. Difference between estimated and real dB value vs the number of measurements a user has performed.

To emphasise this point, Figure 12 shows the difference between the estimated and the real noise level as the users repeatedly perform measurements. Averages and standard deviations are also displayed. This shows that as the expertise increases (number of measurements by the same user - horizontal axis), the errors become closer to zero and deviations from the mean decrease.

**Figure 13:**
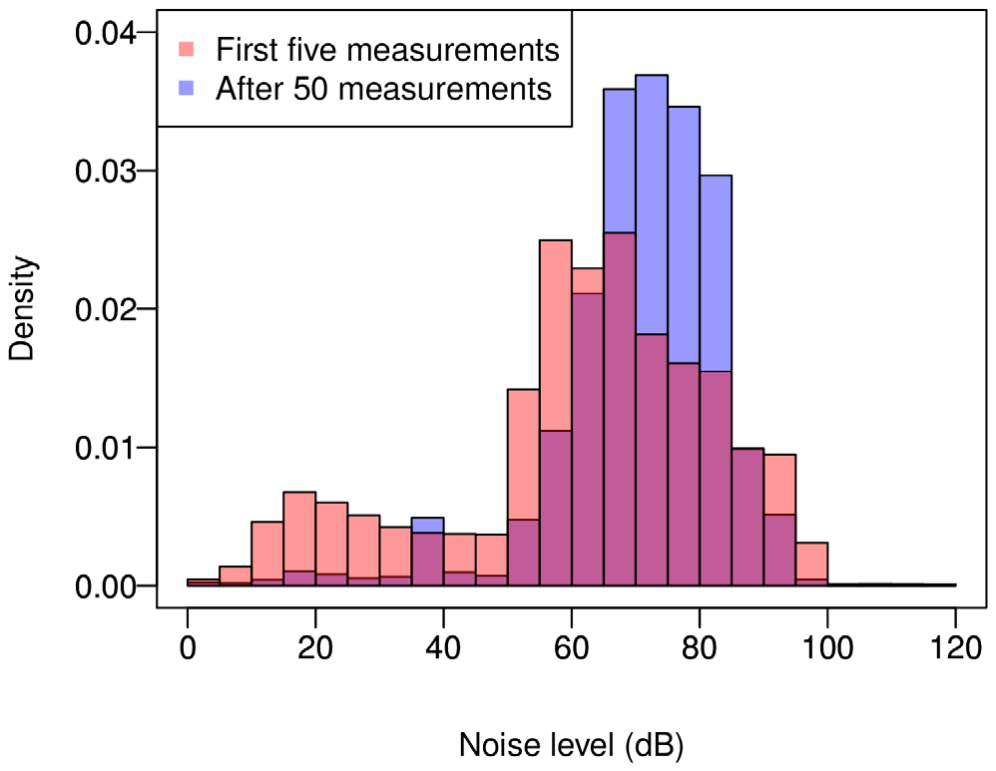
Distribution of measured noise levels. The plot shows the histogram of noise levels for the first measurements performed by users, compared to those performed after some experience is gained (after the 50th measurement).

Considering this, it would be also interesting to see what range of noise is typically measured, and whether this changes in time. Figure 13 displays the distribution of noise levels recorded by users during their first five measurements, compared to those submitted after having already made 50 measurements (43 users have submitted at least 50 measurements). This shows that the noise levels of experienced users are higher than those of novices, indicating that as users become more involved in measurements they tend to concentrate more on areas with high noise levels. This could be on one side due to the users learning how to estimate the higher levels of noise, but also due to an increased interest in documenting higher levels of noise in their area.

**Figure 14:**
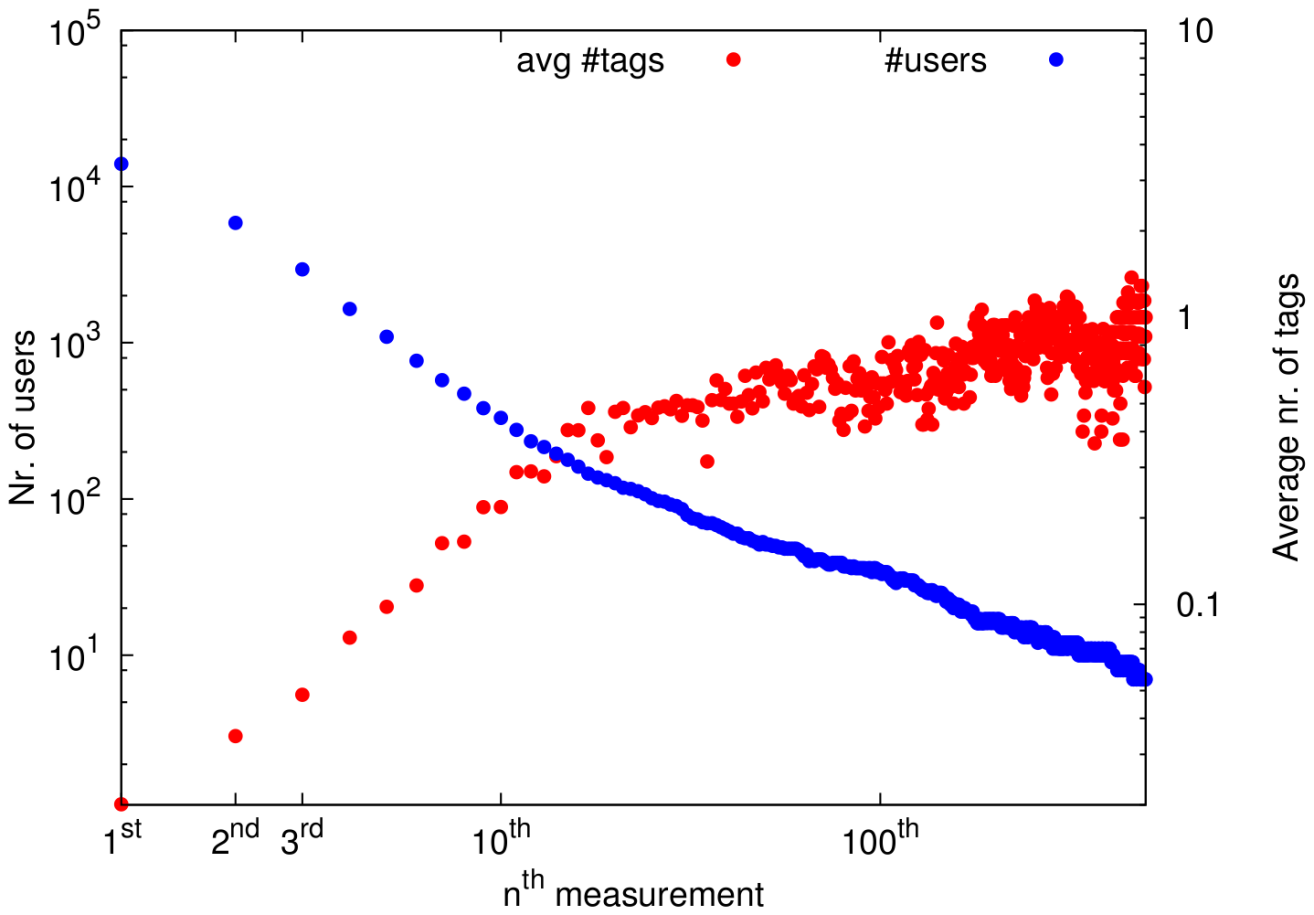
Tagged measurements for different expertise levels. The cumulative number of users submitting at least 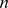 measurements is displayed in blue (left axis legend), while the red points represent the average number of tags used in the 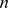-th users' measurement (right axis legend).

A different indicator of user involvement and hence awareness is the amount of tags submitted by users. An increase in repeated application usage would indicate increased involvement in data collection and hence increased awareness. Figure 14 displays the average number of tags per measurement, considering all measurements submitted to the platform, for increasing level of expertise (measurement number). At the same time, the number of users who have passed a certain expertise level is displayed. This shows that as the users perform more measurements, although the number of users here decreases, the average number of tags per measurement tends to increase. This demonstrates an increase in user involvement and dedication to the task, hence in the level of awareness.

**Figure 15:**
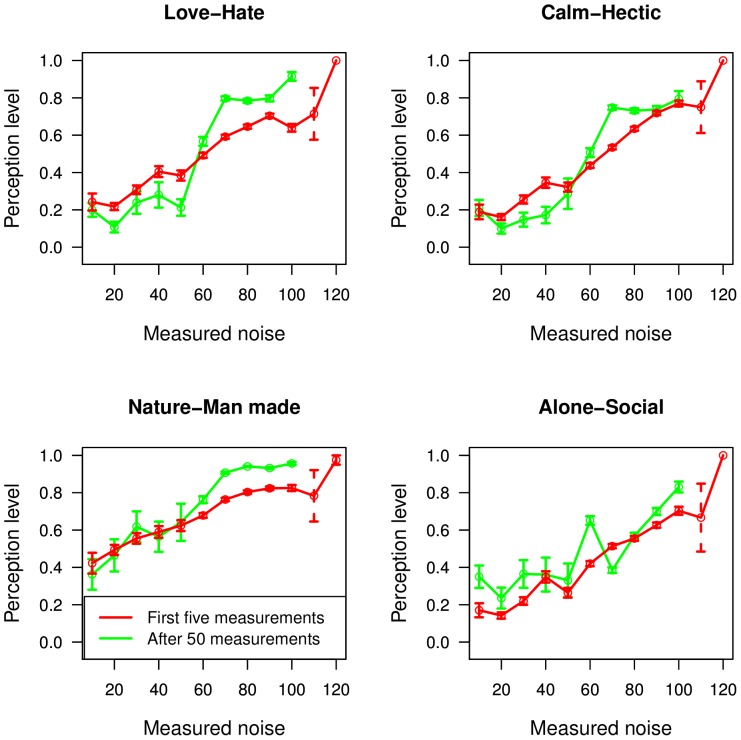
Perception evaluation versus the measured noise level. The red lines display the average evaluation over the first five measurements of all users; the green lines correspond to the average evaluation over the set of all measures taken by users starting from the 50th one.

A further analysis aims to compare the subjective perceptions (Love-Hate, Calm-Hectic, Nature-Man Made, Alone-Social) of the users with the measured noise levels. Out of all measurements performed, 12129 contain perception data. Figure 15, shows how these perceptions depend on the measured noise levels. As expected, the perception values increase with noise. This means that, in general, users ‘Love’ quiet places, finding them a ‘Calm’ environment, while they ‘Hate’ loud ones finding them ‘Hectic’. At the same time, high levels of noise are in general associated with Man-Made and Social environments.

To analyse the change in opinion as the user is exposed to the information from the application, i.e. the real noise level, Figure 15 includes two curves. One shows average perception levels for the first 5 measurements of every user, as a function of noise, while the other shows perceptions for measurements performed after some expertise has been gathered, i.e. more than 50 measurements. The two curves show a different behaviour for novice and expert users, for all perception types except for the Alone-Social evaluation. Specifically, noisy environments are perceived as less pleasant and more artificial as the users become more experienced, while quiet environments as more natural and lovable. A switch between the two possibilities is observed around 55–60 dB, for all three types of perceptions, indicating this as a threshold where noise becomes bothersome. This shows that indeed, exposure to information from the noise application does influence the way in which users perceive the environment. Experienced users have a more stringent evaluation of their environment, and stronger opinions about how much they love or hate the noise levels around. A categorisation of the noise levels appears to emerge, with plateaus visible for high and low levels of noise, when considering data from experienced users. Although it cannot be excluded that experienced users might push the sliders to the extreme right or left edges so to minimize the cognitive effort inherent in judging the quality of noise, the voluntary act of modifying the slider position, by setting it away from the neutral central position, indicates the willingness in conveying a useful information. In that case, we would interpret the pushing of the sliders to the extremes as a conscious act of categorization of experienced users who got more confident with the App. As for the nature-man made indicator, we note that the typical user of our App lives in an urban environment, so that there are fewer samples collected in a natural environment and the error bars associated with the measures are consequently larger, possibly hiding the categorization effect seen in the other indicators at low dB values. The social aspect, however, does not change with repeated usage of the application, since knowing the noise levels does not affect the user's perception of how many individuals there are around. This explains why there is no definite difference between the two curves in Figure 15, lower right pane.

## Conclusions

This paper has presented an analysis of data from participatory noise sensing. For data collection, a mobile application (WideNoise) has been developed and designed to measure noise, while at the same time enabling users to contribute subjective data. Users are called to share their perception/opinion on the level of noise they are measuring in three possible ways. First, they can guess the decibel value the application is going to record, testing their ability in differentiating noise levels and their learning with experience. Second, after the measurement, they can quantify their perception giving a score from zero to one to predefined tags (Love-Hate, Calm-Hectic, Nature-Man Made, Alone-Social). Last, they can make their own annotation, recording any information they wish to share. These three steps of subjective data collection are not mandatory and require different levels of commitment by the users, thus the very exploitation of these possibilities of expression is itself a measure of the user awareness. Overall, these subjective data have been used as a basis for analysis of emerging awareness and learning during measurements.

The WideNoise application has been available for free for smartphone users (most iOS and Android models) and has been widely used around the globe. In addition, several recruiting activities have been performed, mostly in Europe. As data shows, the areas with dedicated campaigns displayed enhanced participation. However, at the same time, these data displayed higher average noise levels, since the dedicated campaigns were mostly motivated by the need to document high levels of noise in residential areas, hence users concentrated on capturing the most noisy periods.

To study awareness and learning, several indicators have been derived from the objective versus subjective data submitted by users, leading to the main findings of this paper:

- Guessed levels of noise, compared to the measured ones, indicate that users learn to estimate the noise level after repeated usage of the application.
- Perception rating is shown to change in time, as users perform more measurements. Hence noisy environments are qualified as more hectic and less lovable by experienced users, compared to novices.
- An increase in the fraction of tags submitted by users was observed as these became more experienced. This suggests an increase in involvement and dedication with time. Together with the change in perception, this indicated an increase in awareness after repeated usage of the WideNoise application.

To the authors knowledge, this is the first study where a throughout parallel investigation of objective and subjective data has been performed, hopefully boosting an increase in awareness toward environmental issues.

Although initial signs of learning and increased awareness have been found already at this level, the usage of the application and evaluation of indicators such as those presented here will be continued in the future. Additionally, an in depth study of several data components is envisioned for future work, such as a semantic analysis of tags, which could give further important insight into both the motivation and opinion of users about their environment.

## Supporting Information

Supplementary Material S1 **This file includes additional details of the WideNoise platform and further analysis of the data, with details not included in the main text.**
(PDF)

## References

[1] Assembly UG (1992) Rio declaration on environment and development. Agenda 21.

[2] World Health Organization, The WHO Centre for Health Development, Kobe, and United Nations Human Settlements Programme (2010) Hidden cities: unmasking and overcoming health inequities in urban settings.

[3] The European Parliament the Council of the European Union (2002) Directive 2002/49/ec of the european parliament and of the council of 25 june 2002 relating to the assessment and management of environmental noise. Official Journal of the European Communities L189 of 18: 12–25.

[4] Passchier-Vermeer W, Passchier WF (2000) Noise exposure and public health. Environmental health perspectives 108: 123. 10698728 10.1289/ehp.00108s1123 PMC1637786

[5] Babisch W, Beule B, Schust M, Kersten N, Ising H (2005) Traffic noise and risk of myocardial infarction. Epidemiology 16: 33–40. 15613943 10.1097/01.ede.0000147104.84424.24

[6] Haines MM, Stansfeld SA, Job RS, Berglund B, Head J (2001) A follow-up study of effects of chronic aircraft noise exposure on child stress responses and cognition. International Journal of Epidemiology 30: 839–845. 11511614 10.1093/ije/30.4.839

[7] Fritschi L, Brown L, Kim R, Schwela D, Kephalopolous S (2011) Burden of disease from environmental noise: Quantification of healthy life years lost in Europe. http://www.who.int/quantifying_ehimpacts/publications/e94888/en/.

[8] Alexander A (2012) Smartphone usage statistics 2012.

[9] Haklay M, Francis L, Whitaker C (2008) Noise Mapping Helps Citizens Take Action.

[10] Cuff D, Hansen M, Kang J (2008) Urban sensing: out of the woods. Communications of the ACM 51: 24–33.

[11] European Environment Agency (2011) NoiseWatch [Mobile application software], Accessed 2013, Oct 29. URL {http://www.eea.europa.eu/mobi}.

[12] Maisonneuve N, Stevens M, Ochab B (2010) Participatory noise pollution monitoring using mobile phones. Information Polity 15: 51–71.

[13] Noise Nuisance Association (2012) Noise Nuisance [Mobile application software], Accessed 2013, Oct 29. URL {http://noisenuisance.org/the-app/}.

[14] Stevens M (2012) Community memories for sustainable societies: The case of environmental noise. Ph.D. thesis, Vrije Universiteit Brussel.

[15] EveryAware Project (Accessed 2013, Oct 29) Available: www.everyaware.eu.

[16] European Council (2006) Decision no 1982/2006/ec of the european parliament and of the council. Official Journal of the European Union.

[17] European Commission (2005) Proposal for a regulation of the european parliament and of the council laying down the rules for the participation of undertakings, research centres and universities in actions under the seventh framework programme and for the dissemination of research results (2007–2013), http://cordis. europa.eu/documents/documentlibrary/82556101en6.pdf.

[18] EveryAware Consortium. Terms and conditions of WideNoise app. Available: http://cs.everyaware.eu/resources/template/noise/files/terms.pdf. Accessed 2013 Oct 29.

[19] EveryAware Consortium. WideNoise app for Android. Available: http://play.google.com/store/apps/details? id = eu.everyaware.widenoise.android. Accessed 2013 Oct 29.

[20] EveryAware Consortium. WideNoise app for iOs. Available: http://itunes.apple.com/app/id657693514. Accessed 2013 Oct 29.

[21] WideTag website. Available: http://www.widetag.com/. Accessed 2013 Oct 29.

[22] Rome testcase website. Available: http://www.everyaware.eu/segiochifaiscienza/. Accessed 2013 Oct 29.

[23] Sano Y, Yamada K, Watanabe H, Takayasu H, Takayasu M (2013) Empirical analysis of collective human behavior for extraordinary events in the blogosphere. Phys Rev E 87: 012805. 10.1103/PhysRevE.87.012805 23410386

[24] Crane R, Sornette D (2008) Robust dynamic classes revealed by measuring the response function of a social system. Proceedings of the National Academy of Sciences 105: 15649–15653. 10.1073/pnas.0803685105 PMC2572957 18824681

[25] Garas A, Garcia D, Skowron M, Schweitzer F (2012) Emotional persistence in online chatting communities. Nature Scientific Reports 402: 1–34. 10.1038/srep00402 PMC3349267 22577512

[26] OSM copyright statement. Available: http://www.openstreetmap.org/copyright. Accessed 2013 Oct 29.

