# Supplementary material for: Awareness and Learning in Participatory Noise Sensing
Source: PLoS One. 2013 Dec 11;8(12):e81638. doi: 10.1371/journal.pone.0081638 (PMC3859489; doi:10.1371/journal.pone.0081638)
Supplement: Supplementary Material S1 — This file includes additional details of the WideNoise platform and further analysis of the data, with details not included in the main text. (PDF) [file pone.0081638.s001.pdf]

Supplementary Material: Awareness and learning in participatory noise sensing

Martin Becker[3,7], Saverio Caminiti[4], Donato Fiorella[5], Louise Francis [2], Pietro Gravino[4], Mordechai (Muki) Haklay [2], Andreas Hotho[3,7], Vittorio Loreto[1,4], Juergen Mueller[6,7], Ferdinando Ricchiuti[5], Vito D.P. Servedio[4], Alina Sîrbu[1,*], Francesca Tria[1]

1 Complex Networks and Systems Lagrange Laboratory, Institute for Scientific Interchange Foundation, Turin, Italy
2 Extreme Citizen Science Research Group, Department of Civil, Environmental and Geomatic Engineering, University College London, United Kingdom
3 University of Würzburg, Germany
4 Physics Department, Sapienza University, Rome, Italy
5 CSP - Innovazione nelle ICT, Turin, Italy
6 University of Kassel, Germany
7 L3S Research Center, Hannover, Germany
∗ **E-mail: alina.sirbu@isi.it**

1 WideNoise Web Platform

The WideNoise web application[1] aggregates, summarizes and illustrates noise related data collected by the WideNoise smartphone application. It provides several statistics for global and personal levels and renders a map for spatial exploration. Additionally, the web application provides useful information about the WideNoise application and its history as can be seen in Figure 1.

Statistics The WideNoise web application provides several statistics on global and personal levels. These statistics help the user to explore and understand the data as well as to observe trends in usage patterns or noise distributions. The statistics (see Figure 2(a)) include but are not limited to: latest recordings and recent average values for different time intervals and locations, user rankings including users with most samples, the most active users, etc. Additionally, perception graphs as well as tag clouds are displayed, characterizing the semantic context of the measurements during several time intervals.

The web application also provides personalised content (see Figure 2(b)): users can access their personal data and statistics via their personal page, e.g. for information on their own measuring behaviour. The personal page also provides a KML[2] export of the user's measurements as an alternative to the *map* visualization.

Map The 'Map' page is one of the most powerful features of the WideNoise web application. For example, a cluster and a grid view are summarizing the noise data providing detailed information on demand [1]. Averages of the measured noise and of the perceptions recorded by the WideNoise smartphone application are available. For registered users a personalized view on the data is provided. Furthermore, a tag cloud characterizes the summarized data by its semantic context. To support social activities, the ability to forward the current view of the map to Twitter or Facebook was introduced. This allows the user to directly share and discuss interesting areas and sample distributions with friends or followers. Another feature of the WideNoise map is the tracking of incoming measurements in real time. Thus, the map connects the user to the ongoing measurement process all over the world.

[1] http://www.widenoise.eu
[2] http://opengeospatial.org/standards/kml

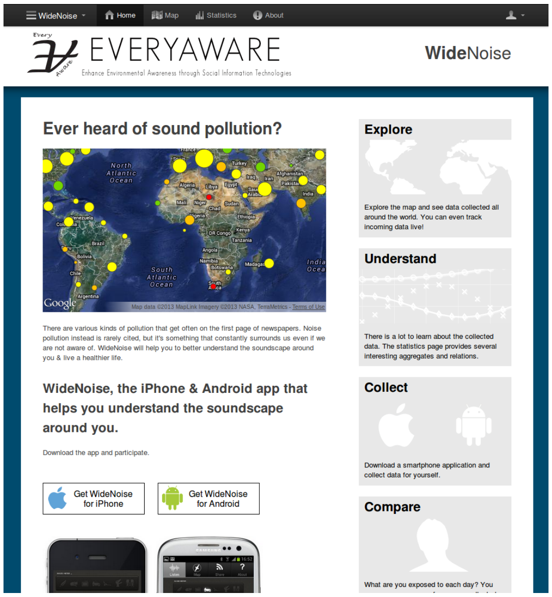

(a) Front page.

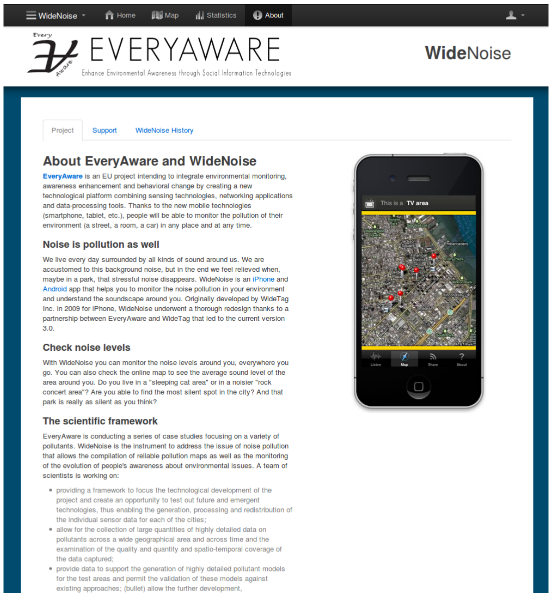

(b) About page.

Figure 1. Snapshots of the Main and About pages in the WideNoise web application.

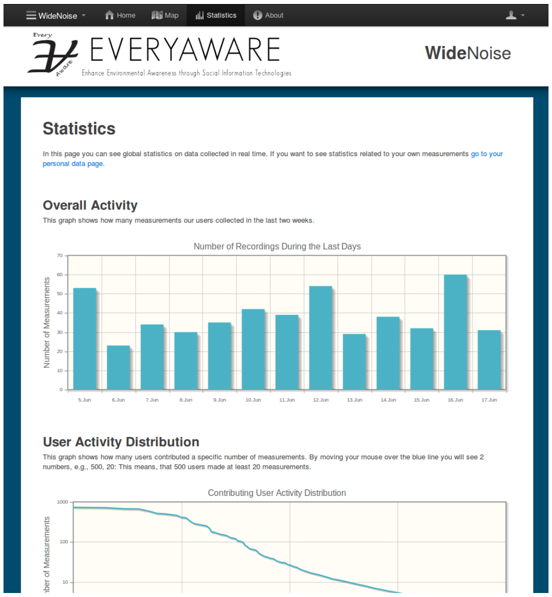

(a) Public.

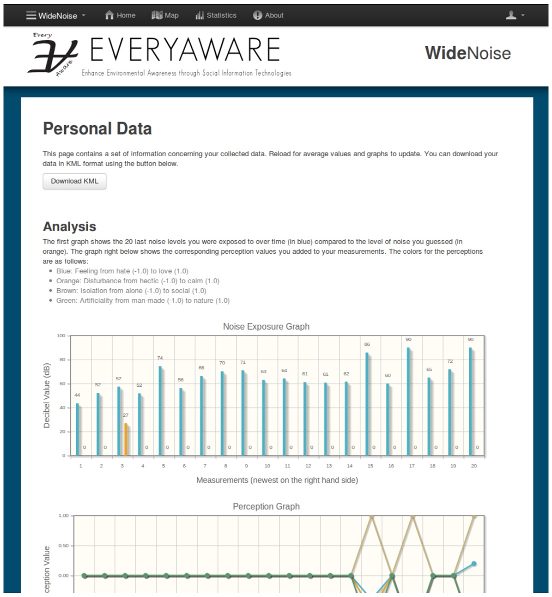

(b) Personal.

Figure 2. General and personal statistics pages hosted by the WideNoise web application.

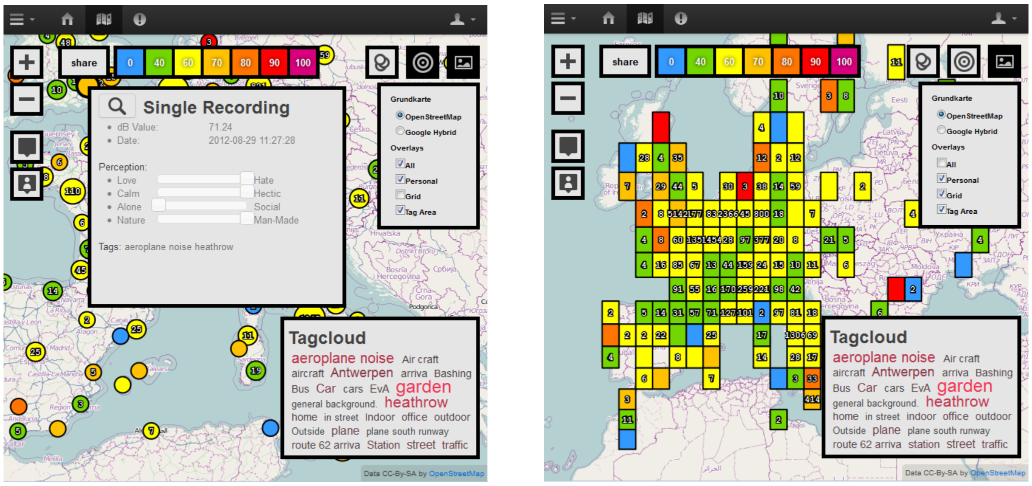

(a) Cluster view with details pop-up..

(b) Grid view.

Figure 3. Map page.

2 Test cases, testing and calibration

2.1 Recruiting activities

One of the research areas within EveryAware is to explore different methods of recruiting participants to carry out participatory sensing activities. As such, we employed a range of approaches to assess their success in engaging the public.

The first was a targeted approach with a captive audience from the Citizen CyberScience summit (16-18th February, 2012). This comprised a relatively small sample of people. The principle behind enlisting this audience was to gain an insight into a specific mode of recruitment and to assess the efficacy of WideNoise. To recruit participants, an email was sent to all the delegates before the start of the conference. This was repeated every morning over the course of the three days. On the first day a short presentation was given inviting people to participate and mini business cards with details about EveryAware and how to install the application were distributed. Delegates were asked to take noise measurements throughout the three days.

The conference was attended by approximately 170 delegates and during this period, and the subsequent days (16th-22nd February), a total of forty-two active Android device installations were made (Number of devices on which the application is currently installed - this excludes any devices where the application was uninstalled). During the three days, 24 unique users installed the Android version of WideNoise for the first time; 17 of these installed the App from within the UK on the first day of conference.

For the second test case we adopted a spatial and contextual approach to recruiting participants by focusing on engaging communities surrounding London Heathrow Airport. To test the effectiveness of mainstream marketing methods we produced an advertisement design, which was used on an advertisement van which drove around the area for a period of five days (26-30th June); banner ads were placed on local websites and advertorials placed in local newspapers. Using more traditional methods, flyers were distributed at key stations around Heathrow and posters were placed in local shop windows. Each one of the advertising channels was assigned a unique website URL to allow click-through tracking monitoring (not the actual number of downloads of the application). Results obtained are detailed in Table 1. These indicate that the most successful method in attracting website visitors and potential application users

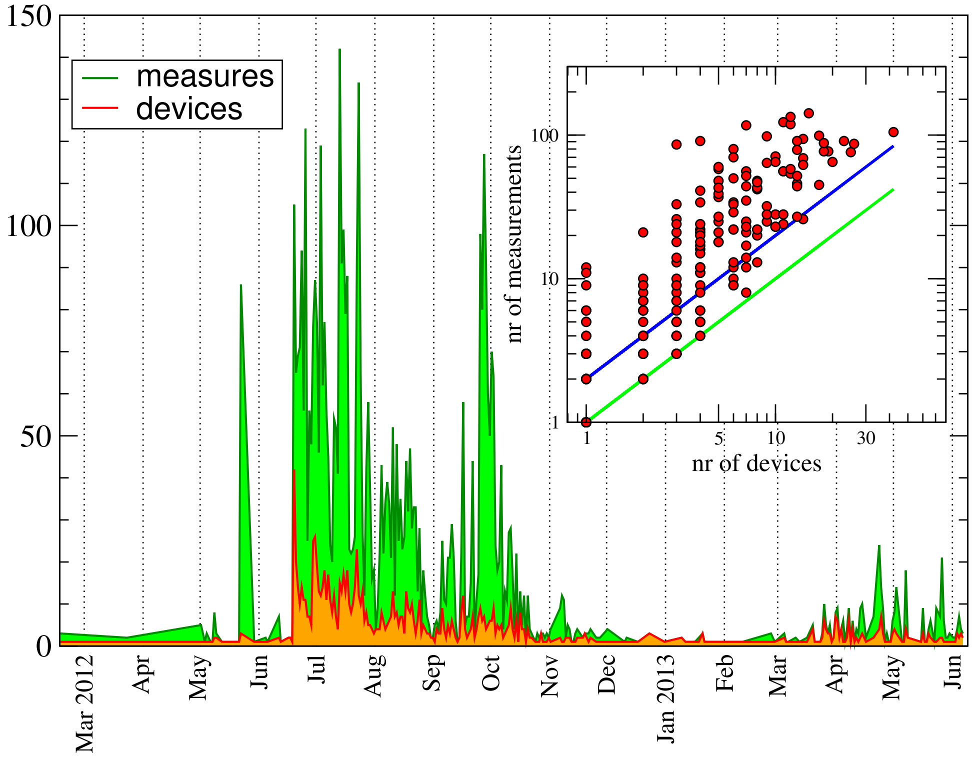

Figure 4. Participation details about the Heathrow test case. The daily activity pertaining the Heathrow test case is shown. Data were filtered out from the whole dataset by restricting to a rectangular area of ca. 7 km (North-South) × 26 km (East-West) around the international airport. The total number of measures collected each day and the number of different recording devices, i.e. the number of users, are shown in green and orange respectively. The scatter plot in the inset relates the number of measures per day to the number of different devices (users). The green and blue lines show those users who on average took one and two samples per day.

was the banner on a local website. This may be due to the increased possibility of an immediate visit on the website through the URL, since the user is already browsing the internet at the moment when they see the ad. For the other advertising methods, the user has to remember the information until they have internet access, which results in lower final number of accesses.

Table 1. Advertising. Results from advertising via different media channels from 6th July to 6th Aug 2012

Advertisement method	Advertisement van (5 days in multiple locations)	Printed advert in local newspaper (once per week for 4 weeks)	Banner advertisement (local website for 4 weeks)	8,000 leaflets handed out at local train stations
No. of unique visitors	21	9	156	13

In addition, news of a launch of monitoring activities in the Heathrow area were circulated using email contacts from HACAN (Heathrow Association for the Control of Aircraft Noise), a campaign organization on behalf of those who suffer because of aircraft flight paths and people were invited to attend a launch event. The combined spatial and contextual focus led to news coverage from the BBC. As a result of all these activities, a total of 285 unique devices were used to take noise recordings over a period of five weeks; 298 have been used in the area since the project inception. This resulted in 3,007 individual recordings over the period. In comparison, a total of 5,518 measurements were taken globally over the same five week period with 1,147 unique devices. Additionally, in April 2013 the Heathrow activities were restarted by the local authorities, yelding additional participation.

A summary of participation in the Heathrow area is shown in Figure 4. This displays daily activity considering all measurements in that area, for the entire period the App has been available (5263 measurements, 419 users). This shows larger activity in the periods when the participation campaigns were active, with more than 100 measurements in some of the days. Comparing this with the overall activity shown in the main text (Figure 2), this explains the slighlty larger activity shown there in the same periods. The inset image also shows the relation between the number of devices and the number of measurements each day, only for the Heathrow area. This shows that, in general, users are more motivated than looking at worldwide data (Figure 3 in main text), with most days generating on average more than 2 measurements per device. However, this average rarely reaches ten measurements per day, and the total number of active devices is seldom larger than 20. This explains why, although these users are more motivated, the worldwide average behaviour does not stand out from the linear trend we have observed.

A third event, again focusing on noise measurement amongst other activities within the context of a specific event, was organized in Rome, Italy, on the 9th June 2012. This event formed part of an activity held within a book store in Rome, ‘Libreria Assaggi’, which is a scientific bookshop. It permitted users to capture noise data in the surrounding streets and visualize the results on a large screen inside the store. In conjunction with this activity, which involved the general public, members of the project team set out to create a systematic noise map of the area surrounding the book store. The event was publicized via Twitter, Facebook and other online web sources, as well as having a dedicated website. It was also advertised in print media throughout the area in the days before the event in order to attract participants from the neighbourhood. Several posters and flyers were placed in areas commonly frequented by local residents.

The location of the bookshop, San Lorenzo is known as a quarter inhabited mainly by university students and with a rich cultural and night life so it seemed a natural choice for the case study. The event itself started at 10.00am and participants were met by members of the EveryAware team as they entered the shop, and were instructed on how to download and use the WideNoise app. Additionally, they were encouraged to register their user details, and then dispatched to various different areas in the location in order to maximise noise measurement coverage. The activity resulted in 688 measurements captured in the Rome area, by a total of 18 distinct users (devices) including 15 members of the general public.

2.2 Evaluation of WideNoise's Performance on Different Mobile Devices

The WideNoise framework, and the EveryAware project in general, is concerned with enhancing participation and awareness of environmental issues, using low cost devices. This inherently means that the measurements performed by the citizens involved are not required to be extremely accurate. However, they should not display such an error that would become confusing. In this section we present tests performed on different devices, analysing the performance of WideNoise as a sound measurement technique.

Tests were carried out in an anechoic sound chamber to develop an understanding of the technical characteristics of the WideNoise Application and mobile devices on which it is deployed. The application

was installed on six different phones (iOS and Android) and these were mounted on a pedestal which also held a calibrated Class 1 reference meter and a Class 2 sound meter. White noise was played through a laptop speaker system into the sound chamber starting with 100 dB(A) and going down in increments of 10 dB(A) to 40 and then finally 31dB(A).

Sound levels were set via the reference device and the corresponding WideNoise reading was taken from each of the mobile phones. This was repeated twice at each decibel step. It was decided to calibrate against dB(A) (measured by the reference devices),even though the WideNoise application does not apply any weighting. This was due to the fact that most other noise related information are usually conveyed in dB(A) [2]. In this way we could provide a comparative data type for the project participants. Table 2 displays the results obtained. The evaluation results suggest that measurements are most accurate for higher levels of noise. For very low noise, even the Class 2 sound meter deviates from the reference, while some models display non monotonic responses (such as the iPhone3). The effective measuring range for WideNoise appears to be thus between 50 dB(A) to 100 dB(A). The level of discrepancy from the reference device within this range was on average of 6.05 dB. Also, it seems that each device deviates in the same direction from the reference on all the effective measuring range, with an exception at the 100 dB level, where the noise level is in most cases (slightly) underestimated by the devices.

Table 2. Device evaluation. Results obtained from the sound chamber evaluation process

Class 1 reference meter	31 dB(A)	40 dB(A)	50 dB(A)	60 dB(A)	70 dB(A)	80 dB(A)	90 dB(A)	100 dB(A)
Class 2 reference meter	35.5	41.8	50.5	60	70	80	90	100
iPod Touch	50	56	64	74	83	93	90	96
iPhone 4	19	35	57	67	80	90	94	96
iPhone 3	42	34	51	64	73	85	93	107
HTC	57	57	60	66	73	88	93	99
HTC Explorer	24	51	61	65	74	89	93	99
Huawei Blaze	51	52	54	57	64	74	84	94

WideNoise was originally developed by WideTag and was then enhanced by the Everyaware team. The application source code inherited uses a hard-coded sound profile based on an unknown device but does not employ a calibration algorithm. Given the results from our test, calibration was not deemed necessary since, although not precise, WideNoise provides a relatively useful indication of the general noise level. Additionally, the fact that the deviations represent a shift on all the dB range compared to the reference is important, since this allows for valid comparisons of different locations. This is very valuable given that the project's aim is to raise people's awareness on acoustic pollution, and identifying the problematic areas is one step in doing so.

3 Data structure and analysis

As of the 7th of June 2013 we collected 41,478 noise samples and registered 13,961 different devices. The measures were collected both by users participating in our organized case studies, and by users who were simply interested in measuring the noise level around them and installed the App on their own. The stream of samples has been continuously growing. The evolution in time of the number of measurements collected is shown in Figure 5. This displays fast growth in the periods of active recruiting activities and dissemination. For instance, the first large increase in the number of measurements appears in February 2012 during the Citizen CyberScience summit in London. The second part of the London Heathrow test case starting in April 2013, cumulated with the publishing of a news article in a local German newspaper,

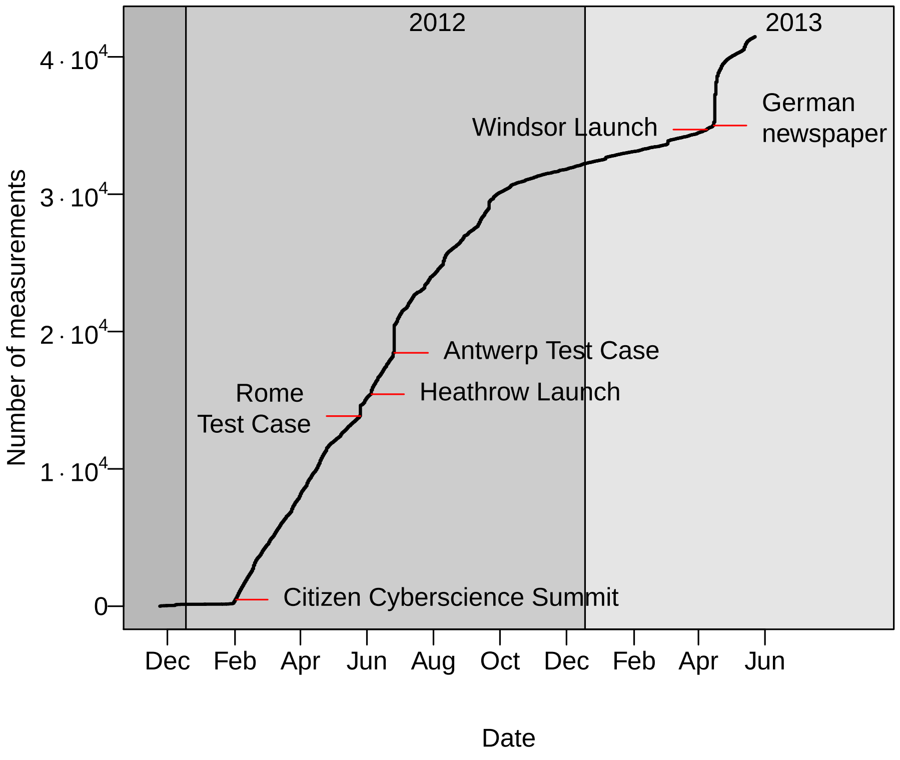

Figure 5. Number of measurements Growth of the total number of measurements collected in time. The main recruiting events and test cases are also indicated.

is also indicated by a fast growth rate. However, this rate decreased in the period when these activities were stopped (e.g. December 2012- February 2013). This indicates the importance of such activities in engaging user participation.

The world-wide noise histogram is shown in Figure 6. It can be noted that there are quite few measures of sound level below 30 dB and above 100 dB. This is mainly due to the fact that smartphone built in microphones are not sufficiently sensitive to measure sound levels below 30 dB and are built to protect the device from very loud sounds, practically limiting the maximum detectable intensity to 100 dB [3]. Moreover, smartphones have to be optimized for human speech and are designed to damp down possible background noise. The figure also shows a discontinuity around 50 dB, where the number of measurements increases a lot. This could be explained by the fact that there is a higher interest of users to measure loud environments, since these are those causing discomfort. Additionally, as we have shown in the main text, there is a high tendency for users to use the application in ‘man-made’ settings rather than natural ones, which are on average noisier.

The aim of the Widenoise App is not only to provide a rough map of noise, but more importantly also to raise people’s awareness on acoustic pollution and possibly trigger behavioural changes. For this reason, the WideNoise application allows users to describe their noise measures with subjective data like

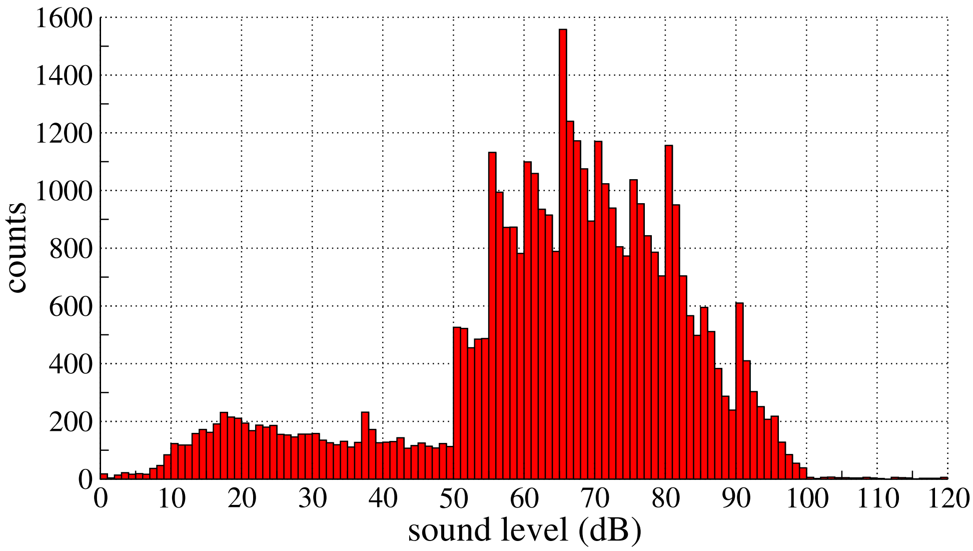

Figure 6. World-wide decibel histogram. Several devices filter out both too mild (below 30 dB) and too loud (above 90 dB) sound levels.

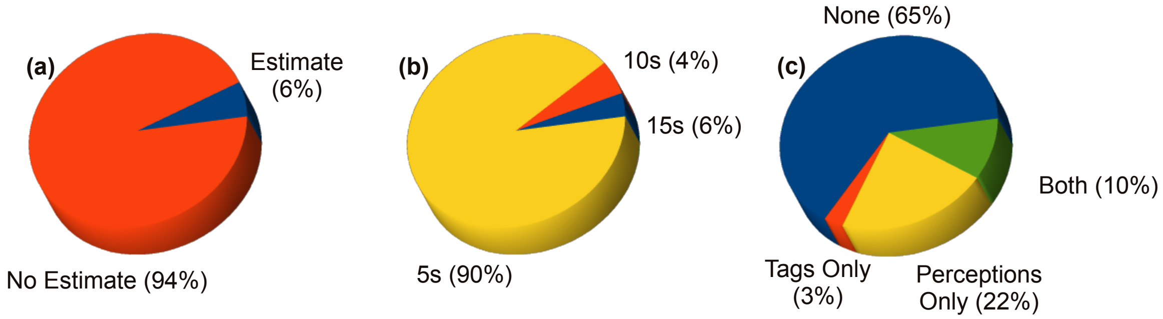

Figure 7. General indicators for collected data. (a) Noise measurements with user estimates and without user estimates; (b) Noise measurements with different recording durations; (c) Noise measurements with different subjective data.

perceptions and tags. The procedure of tagging and noise characterization requires an extra voluntary task that has a cost both in terms of time and cognitive effort. Therefore, we expect that these features have not been used very often. In fact, Figure 7 shows the relation between samples with perceptions only, samples with tags only and samples enriched with both perceptions and tags. A small fraction of the measurements submitted contain subjective data, showing that the application has been mostly used only as a measurement technique. However, some users have been more involved and used the application at its full potential. Based on their activity, an analysis of awareness was presented in the main text. Here we include a few more details to clarify the structure of the data.

Figure 8 shows histograms of how often certain perception values have been used. These include only those measurements where at least one perception had a value different than default (0.5). The figure shows that a large fraction of measurements have default values for perceptions, although one of them is

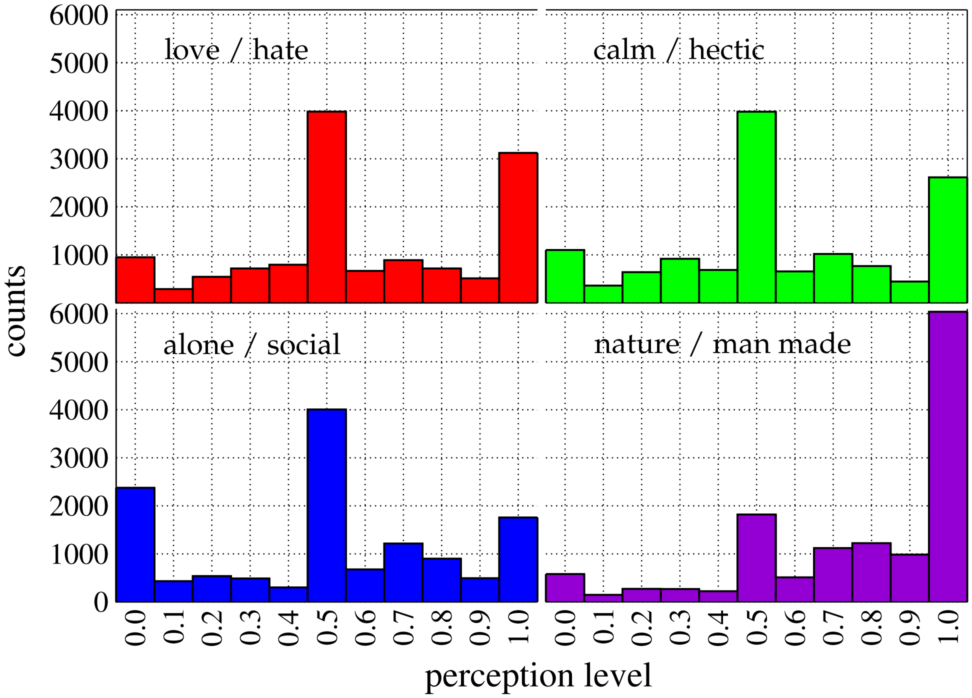

Figure 8. Perception histograms. Counts the frequencies of different perception values in bins of 0.1 for each perception type. The default perception value is 0.5. The histograms are built with those samples where at least one perception was changed from its default value.

used. Secondly, there appears to be a shift towards large perception values, in all categories except for the Alone-Social. This shows again the inclination of users to measure the places they like less or that are more hectic. For the Nature-Man made category, this shift is quite prominent, indicating that most measurements are performed in artificial settings.

Along with perceptions, users may provide tags thus enriching their measures with semantic content. The top 100 tags world-wide are displayed by a tagcloud in Figure 9 (the letter size is proportional to the logarithm of the frequency of that tag). Note how for example *garden*, *heathrow* and *aeroplane noise* are some of the most prominent tags, employed by very motivated users in the frame of the the Heathrow campaign, a large scale case study where WideNoise is used to record noise pollution in a residential region around the Heathrow airport.

Next, we analyse how often subjective data are submitted by users and how their annotation behaviour changes with respect to the WideNoise usage over time. Moreover, by assuming that more motivated users are those who collect many measures, we studied the behaviour of users in time as a function of their motivation. Table 3 shows the number of users who have reached a certain *minimum expertise level*, which we approximately estimate by the number of measurements a user has recorded since the publication of the application. Figure 10 summarizes the analysis of usage over time and motivation described above, for the case of perceptions and tags. Several curves are displayed in this figure, each corresponding to the users who reached at least a certain expertise level. Higher expertise is associated with larger initial motivation. For each minimum expertise level, the corresponding curve displays the evolution of the fraction of annotated measurements (vertical axis) in time. Time is represented here by the number of measurements a user has performed (horizontal axis), i.e. the first measurement is time

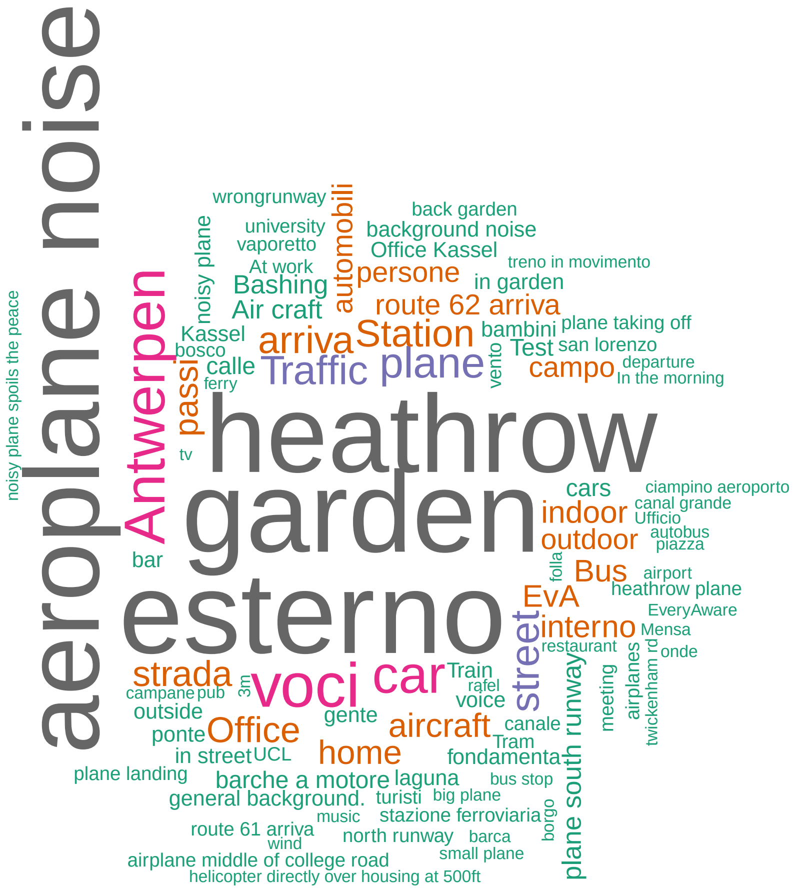

Figure 9. Tagcloud of the 100 most frequent tags used world-wide.

Table 3. Number of users with a minimum expertise level. The expertise level of a user is approximately measured by the total number of his/her recorded measurements. Hence the table shows the number of users submitting at least n measurements.

minimum expertise	50	100	150	200	250	300
number of users	51	34	23	17	15	12

1, the second time 2 and so on. Fractions are computed over time windows of 20 measurements. For each minimum expertise level, the fractions are computed only up to the time when the user has reached that level. For instance, the curve corresponding to a minimum expertise of 200 measurements shows the evolution of the fraction of annotations only up to the 200-th measure, since after this some users included in that category will not have any data associated.

As it can be observed, perceptions are used more often than tags, due to the relative ease of inserting them. The perception dialogue is tightly integrated into the measurement process and it can be used quickly and easily by setting four sliders. The tagging feature, on the other hand, is only offered after

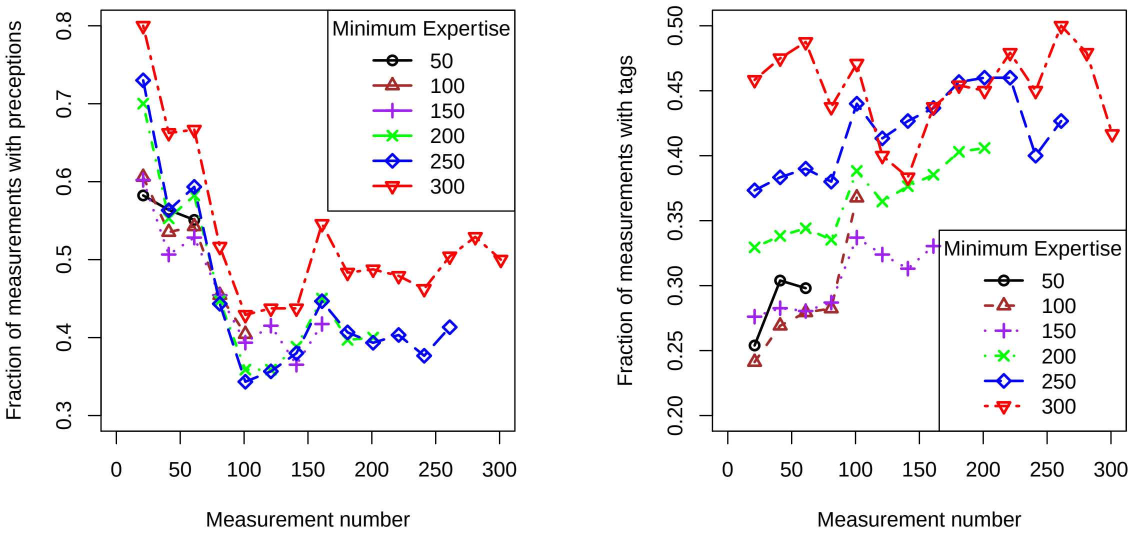

(a) Perceptions. (b) Tags.

Figure 10. Perceptions and tagging trends with increasing usage. The fraction of semantically enriched data over time is shown, i.e., how many measurements are tagged or contain perceptions after using WideNoise for a certain amount of times. The different curves correspond to different user groups, depending on the total number of measurements they performed. Each expertise level includes all users who performed at least as many measurements as the level indicates.

the sample has already been sent and requires the user to type in words, thus, the effort to use tagging is much higher. Perceptions are annotated more often during the first usage phase, especially in the case of highly motivated users, where 80% of measurements have perceptions attached. Over time, i.e. moving along the x-axis, the fraction of annotated perceptions decreases for all user types, reaching a plafond at around 40% and at around 50% for the most expertise users. Tagging, however, follows a different trend, with an increase of the fraction of tagged measurements in time. Since tags require significant effort to be added, this increase suggests an increased involvement of the users, hence more awareness. For the more motivated users, the initial fraction is higher than for the others, thus, the increase appears to be smaller. It is important to note that the fractions computed for the highest expertise levels are computed over a limited set of users, hence have to be considered with caution (see Table 3).

The WideNoise measures were recorded at uncorrelated times and places. While in the main text we show coverage estimators both worldwide and at continent level, here we include details at smaller scales. Table 4 lists time and space coverage values for several areas of interest. Space coverage has been estimated onto a 15 meters by 15 meters grid, where those cells containing at least one measure is considered as “covered”. Time coverage has been calculated by assuming that samples cover 60 seconds, i.e., 30 seconds before and 30 seconds after the recording . We distinguish between two kinds of areas and timespans: continents and areas with higher activity, corresponding to locations where EveryAware test cases have been organised or where we have been locally promoting the application. Our main case studies have been conducted in London, Antwerp and Rome. In London, WideNoise supports an

Table 4. Space and time coverage for main locations.

Location	Nr of measurements	Area covered (km^2)	Total area (km^2)	Fraction	Total time (hours)	Average noise level (dB)	Devices
West London	5263	0.3631	175.9171	0.00206	81.55	74.24	420
London	7338	0.616275	1913.3809	0.00032	111.73	71.87	830
San Lorenzo District, Rome	626	0.0783	0.5127	0.15269	4.29	68.72	23
Rome	1234	0.16425	287.1929	0.00057	12.13	66.13	130
Antwerp Centre	1879	0.1937	2.2363	0.08662	3.38	67.76	18
Antwerp	2214	0.2322	22.0308	0.01053	6.11	67.40	30
Kassel	1369	0.0882	69.4938	0.00126	17.15	57.63	141

ongoing campaign around Heathrow airport against noise pollution caused by air traffic. For the case study in Antwerp Centre a group of participants was split into teams. Each team was assigned to cover a predefined set of streets. The case study in the District San Lorenzo in Rome was designed as a contest. The user who covered the largest area at the end of the day was the winner.

As the table shows, London is the area with the largest activity, with over 15% of the worldwide space and over 20% of the worldwide time coverage. The main factor for this result is the ongoing campaign against noise pollution around Heathrow airport which involves a large number of external users. The location with the largest fraction of the surface covered (over 15%) is the District San Lorenzo in Rome. This result is due to the nature of the case study, which was designed as a contest to cover as much area as possible. Also, the contest area was defined to be relatively smaller, facilitating larger coverage.

It is important to note how, in general, measurements coming from test case areas (West London, San Lorenzo, Antwerp Centre) report on average higher noise than the corresponding city averages (London, Rome, Antwerp). This indicates that users involved in test cases are oriented towards uncovering areas and sources of noise. In the Heathrow test case this is obvious, since users got involved to monitor noise generated by airplanes, which is a nuisance in the area. However, case studies resulted in higher noise averages at other locations as well. Given that all test cases were performed in Europe, this could partially explain why the data indicate Europe to be the loudest continent so far.

To understand the details of the coverage better and to uncover trends in user participation, it is interesting to study how user activity and average noise levels are distributed over certain time intervals such as “day of the week” or “hour of the day” .

Figure 11 shows the measured noise levels (averages) over different hours of the day. Noise levels increase sharply in the morning and then slowly until the evening, when they decrease to low dB values during the night. The corresponding distribution of the number of measurements (worldwide) and the number of devices (users) used are also included. The number of users increases in the morning and is quite stable until early evening when it increases slightly (possibly due to social outdoors activities). The number of measurements, however, displays two peaks late morning and afternoon (corresponding to coffee breaks at work). This indicates that although at these times user numbers do not increase, those devices that are used perform more measurements than at other times of the day. This could be due again to the social effect (people discuss noise pollution and make repeated measurements).

A similar analysis has been performed for different days of the week (see Figure 12). The distribution of sample counts and devices shows a large activity on Tuesdays, with other days of the week more inactive and an increase in sample counts over the weekend. The Tuesday peak can be explained by the fact that, by chance, many of the test cases and activities were organised on Tuesdays. This was the

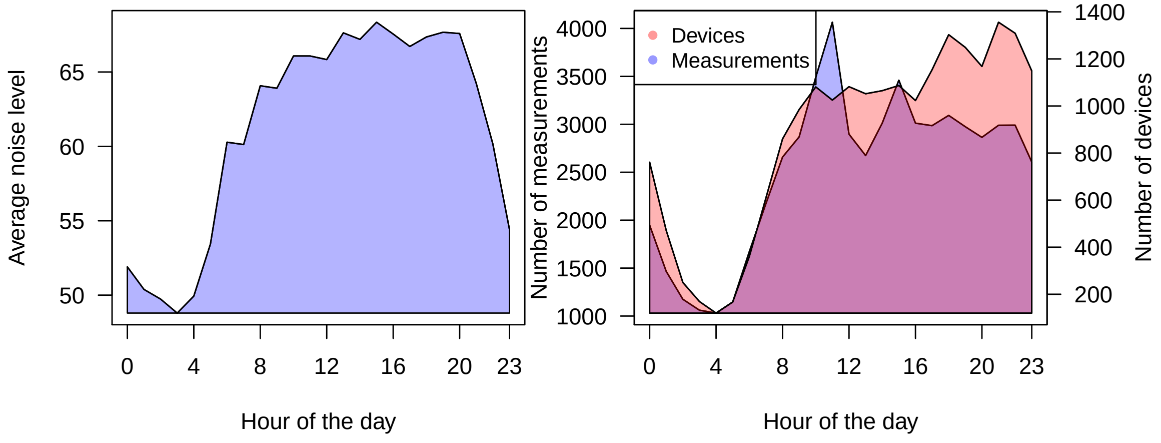

Figure 11. Hourly activity. Worldwide average noise levels and corresponding number of measurements and devices at different hours of the day.

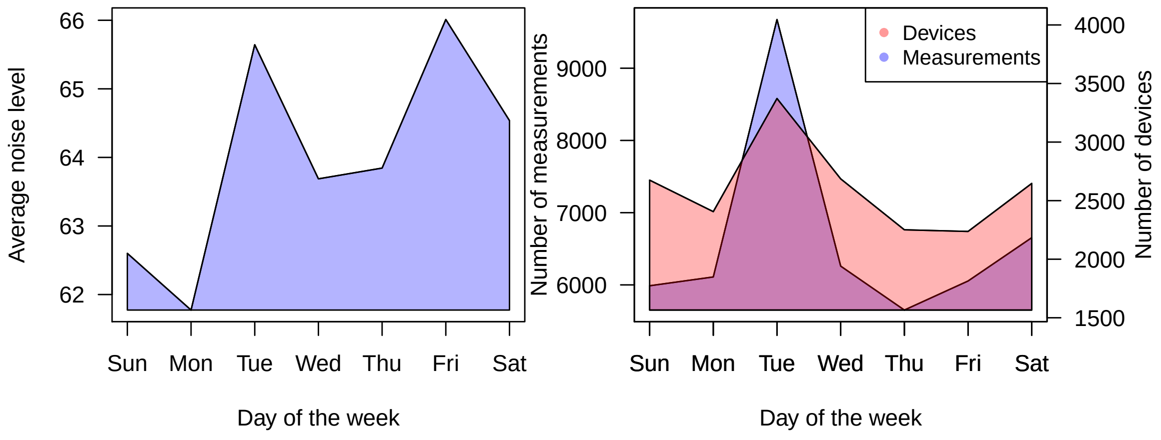

Figure 12. Daily activity. Worldwide average noise levels and corresponding number of measurements and devices during different days of the week.

case for the Antwerp test case and the launch of the Heathrow campaign. Additionally, the peak activity in the Windsor area was on a Tuesday as well. The higher levels of activity on Fridays and during the weekends are, on the other hand, to be expected, since that is the time for more social and outdoors activities. The noise levels displayed indicate Tuesdays and Fridays as the most noisy days. Since samples are rather concentrated on Tuesdays, as we have already mentioned, this peak could be an artefact. The peak on Friday could be explained by extra traffic for commuting and noise related to social activities in the evening.

References

1. Shneiderman B (1996) The eyes have it: A task by data type taxonomy for information visualizations. In: Symposium on Visual Languages, Boulder, CO, USA – 3-6 September, 1996. Proceedings. IEEE, pp. 336–343. doi:10.1109/VL.1996.545307.

2. Pierre Jr RLS, Acoustics R, Maguire DJ, Automotive CS (2004) The impact of a-weighting sound pressure level measurements during the evaluation of noise exposure. In: Conference NOISE-CON. pp. 12–14.

3. Stevens M (2012) Community memories for sustainable societies: The case of environmental noise. Ph.D. thesis, Faculty of Science, Vrije Universiteit Brussel, Brussels, Belgium. URL `http://brussense.be/phd-matthias`.
